# Convergent Neuroimaging and Molecular Signatures in Mild Cognitive Impairment and Alzheimer’s Disease: A Data-Driven Meta-Analysis with *N* = 3,118

**DOI:** 10.1007/s12264-024-01218-x

**Published:** 2024-06-01

**Authors:** Xiaopeng Kang, Dawei Wang, Jiaji Lin, Hongxiang Yao, Kun Zhao, Chengyuan Song, Pindong Chen, Yida Qu, Hongwei Yang, Zengqiang Zhang, Bo Zhou, Tong Han, Zhengluan Liao, Yan Chen, Jie Lu, Chunshui Yu, Pan Wang, Xinqing Zhang, Ming Li, Xi Zhang, Tianzi Jiang, Yuying Zhou, Bing Liu, Ying Han, Yong Liu, The Alzheimer’s Disease Neuroimaging Initiative, The Multi-Center Alzheimer’s Disease Imaging (MCADI) Consortium

**Affiliations:** 1 School of Artificial Intelligence, University of Chinese Academy of Sciences, Beijing, 100049 China; 2 Brainnetome Center and National Laboratory of Pattern Recognition, Institute of Automation, Chinese Academy of Sciences, Beijing, 100190 China; 3 Department of Radiology, Qilu Hospital of Shandong University, Ji’nan, 250063 China; 4 Department of Neurology, the Second Affiliated Hospital of Air Force Medical University, Xi’an, 710032 China; 5 Department of Radiology, Chinese PLA General Hospital, Beijing, 100853 China; 6 Department of Radiology, the Second Medical Centre, National Clinical Research Centre for Geriatric Diseases, Chinese PLA General Hospital, Beijing, 100853 China; 7 School of Artificial Intelligence, Beijing University of Posts and Telecommunications, Beijing, 100191 China; 8 Department of Neurology, Qilu Hospital of Shandong University, Ji’nan, 250063 China; 9 Department of Radiology, Xuanwu Hospital of Capital Medical University, Beijing, 100053 China; 10 Branch of Chinese, PLA General Hospital, Sanya, 572013 China; 11 Department of Neurology, the Second Medical Centre, National Clinical Research Centre for Geriatric Diseases, Chinese PLA General Hospital, Beijing, 100853 China; 12 Department of Radiology, Tianjin Huanhu Hospital, Tianjin, 300222 China; 13 Department of Psychiatry, People’s Hospital of Hangzhou Medical College, Zhejiang Provincial People’s Hospital, Hangzhou, 310014 China; 14 Department of Radiology, Tianjin Medical University General Hospital, Tianjin, 300070 China; 15 Department of Neurology, Tianjin Huanhu Hospital, Tianjin, 300222 China; 16 Department of Neurology, Xuanwu Hospital of Capital Medical University, Beijing, 100053 China; 17 Key Laboratory of Animal Models and Human Disease Mechanisms of the Chinese Academy of Sciences & Yunnan Province, Kunming Institute of Zoology, Chinese Academy of Sciences, Kunming, 650201 Yunnan China; 18 State Key Lab of Cognition Neuroscience & Learning, Beijing Normal University, Beijing, 100875 China; 19 National Clinical Research Center for Geriatric Disorders, Beijing, 100053 China; 20 Center of Alzheimer’s Disease, Beijing Institute for Brain Disorders, Beijing, 100053 China

**Keywords:** Alzheimer’s disease, Structural magnetic resonance imaging, Meta-analysis, Brain atrophy, Gene set enrichment analysis

## Abstract

The current study aimed to evaluate the susceptibility to regional brain atrophy and its biological mechanism in Alzheimer’s disease (AD). We conducted data-driven meta-analyses to combine 3,118 structural magnetic resonance images from three datasets to obtain robust atrophy patterns. Then we introduced a set of radiogenomic analyses to investigate the biological basis of the atrophy patterns in AD. Our results showed that the hippocampus and amygdala exhibit the most severe atrophy, followed by the temporal, frontal, and occipital lobes in mild cognitive impairment (MCI) and AD. The extent of atrophy in MCI was less severe than that in AD. A series of biological processes related to the glutamate signaling pathway, cellular stress response, and synapse structure and function were investigated through gene set enrichment analysis. Our study contributes to understanding the manifestations of atrophy and a deeper understanding of the pathophysiological processes that contribute to atrophy, providing new insight for further clinical research on AD.

**Supplementary Information:**

The online version contains supplementary material available at 10.1007/s12264-024-01218-x.

## Introduction

Alzheimer’s disease (AD) is a chronic neurodegenerative disease characterized by progressive memory loss and cognitive impairment and is the predominant type of dementia. Neuron loss is one of the most predominant biomarkers of AD [1, 2], associated with the atrophy of gray matter. Studying brain morphology using structural magnetic resonance imaging (sMRI) provides a powerful way to screen and diagnose AD *in vivo* [3, 4]. Gray matter volume (GMV) and cortical thickness (CT) are the most commonly used measurements based on sMRI images, which respond to changes from different aspects [5–7]. It is important to establish the standard atrophy mapping on AD to reflect the common mechanism of neuron loss; however, this has not been consistent between studies due to the small sample size from a single site.

Previous studies have conducted literature-based meta-analyses to investigate the atrophy pattern in AD [8, 9]. The literature-based meta-analysis also has potential limitations, such as publication bias, heterogeneity in the analysis steps, and statistical criteria of included studies [10]. Nevertheless, by analyzing the raw data from different sites with state-of-the-art steps, a data-driven meta-analysis allowed a more robust detection of case-control differences [11–14]. Hence, we expected to obtain reliable, systematic results of brain alteration using data-driven meta-analysis with a significantly larger sample.

One of the most recognized hypotheses in AD is the neurotoxic accumulation of amyloid beta (Aβ), which leads to neuron death and atrophy [15]. In addition, other underlying biological changes, such as defective protein quality control and degradation pathways, dysfunctional mitochondrial homeostasis, stress granules, and maladaptive innate immune responses, have been thought to cause proximal changes in brain structure and function in AD [16]. However, the underlying biological mechanisms behind the atrophy in AD remain elusive [17, 18]. Imaging transcriptomics analysis is a rapidly emerging field that combines magnetic resonance imaging (MRI) and genetic profiles, which has the potential to identify atrophy-related genes and pathways [19, 20]. Therefore, we hypothesized that linking the atrophy pattern with the transcriptomics patterns could offer a comprehensive multimodal perspective for understanding the central nervous system abnormalities in AD.

The main aim of the current study was to systematically evaluate the susceptibility of regional brain atrophy and its biological mechanism. Firstly, we applied data-driven meta-analysis to investigate reliable, systematic results by combining region of interest (ROI) features using a large sample with 3,118 subjects from 3 multi-site databases (a total of 23 sites). Then we systematically evaluated the genetic and molecular basis of the alterations in brain structure in MCI and AD using spatial whole-brain gene and neurotransmitter mapping.

## Materials and Methods

### Data Collection and Preprocessing

The Medical Ethics Committee of the Institute of Automation, Chinese Academy of Sciences, approved this study. T1-weighted sMRI data were acquired from three multi-site datasets: our in-house Multi-Center Alzheimer’s Disease imaging dataset (MCAD) [21], the Alzheimer’s Disease Neuroimaging Initiative dataset (ADNI) [22], and the European Diffusion Tensor Imaging (DTI) Study on Dementia dataset (EDSD) [23]. The MCAD studies were approved by the medical ethics committees of the local hospitals, and all the subjects or their legal guardians gave written consent. All participants underwent a battery of neuropsychological tests and fulfilled specific inclusion criteria. Images were scanned on eight scanners with identical and stringent standards (Table S1). Race and ethnicity information for ADNI and EDSD is detailed in their dataset description, and the in-house MCAD dataset consists of Asians. The MCAD dataset includes data from 8 sites. The EDSD dataset includes data from 12 sites. For the ADNI dataset, we considered the different phases of the ADNI (ADNI1, ADNI2, and ADNI3) as different sub-datasets since the sample sizes of their original sites were relatively small. Detailed subject inclusion and diagnostic criteria, machine acquisition parameters, and other information can be found in Supplementary Material 1. The data included patients with MCI, AD, and normal controls (NC) of similar age. Patients with other psychiatric disorders were excluded. We collected 3,168 subjects with baseline imaging data for data preprocessing.

First, two experienced researchers (Y.L. and X.K.) performed a visual check to exclude subjects with significant noise. Then all the images were preprocessed using the standard steps of the Computational Anatomy Toolbox 12 (CAT12, http://www.neuro.uni-jena.de/cat/) segmentation process. After the segmentation, the gray matter images with a voxel size of 1 mm × 1 mm × 1 mm in Montreal Neurological Institute (MNI) space were generated. Then we applied a Gaussian filter with 4 mm full-width at half maximum to the gray matter image. We applied the Brainnetome atlas [24] to smooth gray matter images to calculate 246 ROI GMVs. And 210 ROI mean CTs of the Brainnetome atlas were calculated by CAT12 during the segmentation process. Fifty subjects with low quality (resolution, noise, bias, image quality rating (IQR) rated by CAT12 segmentation, 60% as threshold) were excluded to ensure the reliability of the analysis [25]. The remaining 3,118 subjects were included.

Furthermore, we collected 1,003 Aβ (AV45) and 912 ^18^F-fluorodeoxyglucose (FDG) positron emission tomography (PET) images from the ADNI for neuropathophysiological analysis. These images were first registered to the corresponding T1 image and from there to MNI space. Then, each participant’s PET value (Aβ and FDG) was normalized by dividing it by the PET value of the cerebellum and applying the Brainnetome atlas to calculate 246 average PET values of each ROI.

### Statistical Analysis of General Atrophy Patterns

We applied data-driven meta-analysis to analyze the neuroimaging features, measure the degree of atrophy in each ROI, and construct a whole-brain atrophy pattern. The null hypothesis of equality in the gray matter volume (cortical thickness) between the AD and the NC groups was tested independently for each site. Before we included these features in the meta-analysis, the effects of age, sex, and total intracranial volume were removed using linear regression on each site.

Next, we used Cohen’s *d* to measure the effect size for each site and estimated the weight for each site using the random model and inverse-variance method. The summary effect size was derived from the weighted sum of the effect sizes of the sites [26]. Gray matter (volume, thickness) in each ROI (or voxel) was associated with an effect size (Cohen’s *d*) for each site. Cohen’s *d*, or standardized mean difference, one of the most common ways to measure effect size, is calculated by the following formula:

$$d = \frac{\overline{{x}_{1}}-\overline{{x}_{2}}}{s}$$

where $\overline{{x}_{1}}$ and $\overline{{x}_{2}}$ are the mean values of the feature in group 1 and group 2 in the specified site. The pooled standard deviation $s$ is calculated as $\sqrt{\frac{\left({n}_{1}-1\right){{s}_{1}}^{2}+\left({n}_{2}-1\right){{s}_{2}}^{2}}{{n}_{1}+{n}_{2}-2}}$. Where ${n}_{1}$ and ${n}_{2}$ are the number of subjects in group 1 and group 2, ${s}_{1}$ and ${s}_{2}$ are the standard deviations of the feature in group 1 and group 2, and $s$ is the pooled standard deviation.

For each ROI, the summary effect size was calculated by combining the effect size of each site using a random model and inverse variance method. The weight for each site is calculated using the following formula:

$${{\text{weight}}}_{{\text{random}}} = \frac{1}{v+{\tau }^{2}}$$

where ${{\text{weight}}}_{{\text{random}}}$ is the weight of the random model for each site in the meta-analysis, $v$ is within-site variance calculated as $\frac{{n}_{1}+{n}_{2}}{{n}_{1} \times {n}_{2}}+\frac{{d}^{2}}{2({n}_{1}+{n}_{2})}$, ${\tau }^{2}$ is the between-site variance calculated as $\frac{Q-{\text{df}}}{C}$, $Q$ is calculated as $\sum \frac{\sqrt{d-\overline{d}}}{v}$, ${\text{df}}$ is the degree of freedom, *C* is calculated as $\sum {{\text{weight}}}_{{\text{fixed}}}-\frac{\sum \sqrt{{{\text{weight}}}_{{\text{fixed}}}}}{\sum {{\text{weight}}}_{{\text{fixed}}}}$, and the ${{\text{weight}}}_{{\text{fixed}}}$ is calculated as $\frac{1}{{\text{variance}}}.$

After the effect size and weight of each site were calculated, we calculated the summary effect size by the weighted sum method:

$${d}_{{\text{summary}}} = \frac{\sum_{i}^{k}{{\text{weight}}}_{i} \times {d}_{i}}{\sum_{i}^{k}{{\text{weight}}}_{i}}$$

where $k$ is the total number of sites, ${{\text{weight}}}_{i}$ is the weight of site $i (\text{computed with a random model}),$ ${{\text{and}} \,d}_{i}$ is the effect size of the site $i$.

Finally, a *Z*-value to test the null hypothesis that the summary weighted effect is zero was computed using:

$$Z = \frac{{d}_{{\text{summary}}}}{{{\text{SE}}}_{{\text{summary}}}}$$

where ${{\text{SE}}}_{{\text{summary}}}$ is the estimated standard error of the summary effect, calculated as $\sqrt{\frac{1}{\sum_{i}^{k}{{\text{weight}}}_{i}}}$.

For a two-tailed test, the *P-*value is given by:

$$P = 2 \times [1-\Phi \left(\left|Z\right|\right)]$$

where $\Phi \left(\left|Z\right|\right)$ is the standard normal cumulative distribution. The Bonferroni correction was used to correct for multiple comparisons across all the measures.

Besides meta-analysis, we also measured the association between gray matter features in each brain region and cognitive scores using Pearson correlation coefficients, and we compared it with atrophy patterns.

### Spatial Alignment to Neuropathophysiological Features

We investigated the mechanisms underlying AD atrophy using the human brain gene transcriptome data extracted from the Allen Human Brain Atlas (AHBA) [27, 28]. We matched the gene expressions for each ROI using the Abagen toolbox [29, 30], resulting in 246/210 × 15,633 matrices for the GMV/CT-based analysis. One partial least squares (PLS) regression algorithm, the statistically inspired modification of the partial least squares (SIMPLS), was applied to investigate how genetic variance can explain brain structural alterations [19, 31]. The ranked gene list obtained using principal PLS weights (PLS1) was fed into the online tool WebGestalt [32] to identify the functional enrichment by gene set enrichment analysis (GSEA) [33]. A significance level of *P*_FDR_ <0.05 was applied for all enrichment analyses.

To further elucidate our GSEA results, we also introduced PET/single-photon emission computed tomography (SPECT) maps of 12 neuropathophysiological features from unrelated control groups for spatial alignment [34] (further details in Supplementary Material 1). The correlation was calculated between the effect sizes of the ROI atrophy and regional mean PET/SPECT values. Furthermore, we extracted each ROI’s sum for Aβ and FDG PET image analysis and applied case-control *t*-tests between groups. Then we calculated the correlation between the effect sizes of the ROI atrophy based on all subjects and the ADNI PET ROI *t*-values, resulting in a single measure for assessing the relationship between the altered brain structure and the altered Aβ or FDG in AD. We also used ADNI subjects with both T1 images and PET images to analyze the correlation between gray matter features and the distribution of Aβ/FDG in brain regions.

### Robustness and Reliability Analysis

To verify the robustness and reliability of the results, we applied further validations for each analysis. For the meta-analysis, we first analyzed the correlation between the effect sizes of the individual sites and their correlation with the summary effect size. We also performed data-driven meta-analyses on the original ROI values without removing covariates, and meta-analyses based on data controlled for age^2^. We also applied additional meta-analyses within a single dataset. To avoid atlas-induced bias, we used alternative brain atlases such as the automated anatomical labelling (AALv3) atlas [35] and Schaefer atlas 1,000 [36] to calculate ROI values for meta-analysis. Furthermore, we applied the bootstrapping strategy to the meta-analyses and sampled 80% of the subjects for each site to calculate the effect size in each iteration. We also applied voxel-wise or vertex-wise meta-analyses to supplement the ROI-wise analysis on gray matter images and cortical features. Further, we used neuroCombat [37] to harmonize gray matter features to mitigate site effects, performed *t*-tests based on the harmonized data, and compared them with meta-analysis results.

To account for spatial autocorrelation (SA), ensure statistical test validity, and avoid false-positive results, we used atrophy patterns to generate 5,000 SA-preserving surrogate maps as null models using BrainSMASH [38, 39]. We applied SIMPLS analysis between each surrogate map and AHBA gene expression and validated the variance explained by PLS1. Further, we randomly took 500 surrogate map PLS1s for GSEA and compared the enrichment pathways and their enrichment scores with the results in the main analysis. The significance is calculated as ${P}_{{\text{SA}}} = \frac{{{\text{Count}}}_{{\text{Greater}}}}{{{\text{Count}}}_{{\text{All}}}}$, where ${{\text{Count}}}_{{\text{Greater}}}$ is the times that the surrogate’s enrichment score is greater than the enrichment scores of the primary analysis. ${{\text{Count}}}_{{\text{All}}}$ is the number of times that surrogate GSEA was performed, in this case, 500 times.

For the atrophy-PET correlation analysis, we generated 5,000 SA-preserving surrogate maps for each atrophy pattern as null models. The significance is calculated as ${P}_{{\text{Corr}}\_{\text{SA}}} = \frac{{{\text{Count}}}_{{\text{Greater}}}}{{{\text{Count}}}_{{\text{All}}}}$, where ${{\text{Count}}}_{{\text{Greater}}}$ is the number of times that the surrogate’s absolute correlation coefficient is greater than that of the primary analysis. ${{\text{Count}}}_{{\text{All}}}$ is the number of times that surrogate correlation was performed, in this case, 5,000 times.

## Results

### Participants

A total of 834 AD subjects [mean ± SD age, 71.73 ± 8.87 years; 466 female (56%); 368 male (44%); Mini-Mental State Examination (MMSE), 19.63 ± 5.65], 1,135 MCI subjects [71.92 ± 8.26 years; 509 female (45%); 626 male (55%); MMSE, 26.70 ± 2.61], and 1,149 NC subjects [70.05 ± 8.02 years; 626 female (54%); 523 male (46%); MMSE, 28.90 ±1.28] were included in the study. Detailed demographic features, neuropsychological test scores, and total tissue measures can be found in Table 1, the subject counts for each site in Fig. S1, and the image quality ratings in Fig. S2. Fig. S3 provides detailed demographic information of the included subjects with PET images from ADNI.

**Table 1:** Detailed subject information for each dataset.

	MCAD			ADNI			EDSD		
	NC	MCI	AD	NC	MCI	AD	NC	MCI	AD
Male/female	147/188	138/162	154/245	278/322	412/283	147/130	98/116	76/64	67/91
Age	64.71(8.86)	68.49(9.26)	69.23(9.15)	73.48(6.11)	73.23(7.66)	74.86(7.75)	68.79(6.19)	72.80(6.61)	72.56(8.06)
MMSE	28.60(1.58)	25.15(3.49)	16.67(5.96)	29.09(1.09)	27.46(1.82)	23.14(2.11)	28.82(1.14)	26.28(2.08)	20.96(5.03)
CSF	378(77)	419(94)	459(96)	392(87)	432(96)	473(103)	355(73)	431(82)	439(71)
GMV	590(53)	563(59)	524(63)	549(53)	532(54)	499(54)	551(53)	516(55)	478(56)
WMV	492(54)	471(56)	442(57)	478(62)	477(64)	455(62)	487(63)	458(64)	432(67)
TIV	1,463(140)	1,456(146)	1,430(141)	1,425(154)	1,448(156)	1,436(176)	1,398(149)	1,413(152)	1,358(152) )

Data are presented as the mean (SD). MMSE, Mini-Mental State Examination; CSF, cerebrospinal fluid; GMV, gray matter volume; WMV, white matter volume; TIV, total intracranial volume; MCAD, Multi-Center Alzheimer’s Disease Imaging; ADNI, Alzheimer’s Disease Neuroimaging Initiative; EDSD, European Diffusion Tensor Imaging Study On Dementia; NC, normal control; MCI, mild cognitive impairment; AD, Alzheimer’s disease.

### General Atrophy Pattern for Cognitively Impaired States

**Fig. 1:**
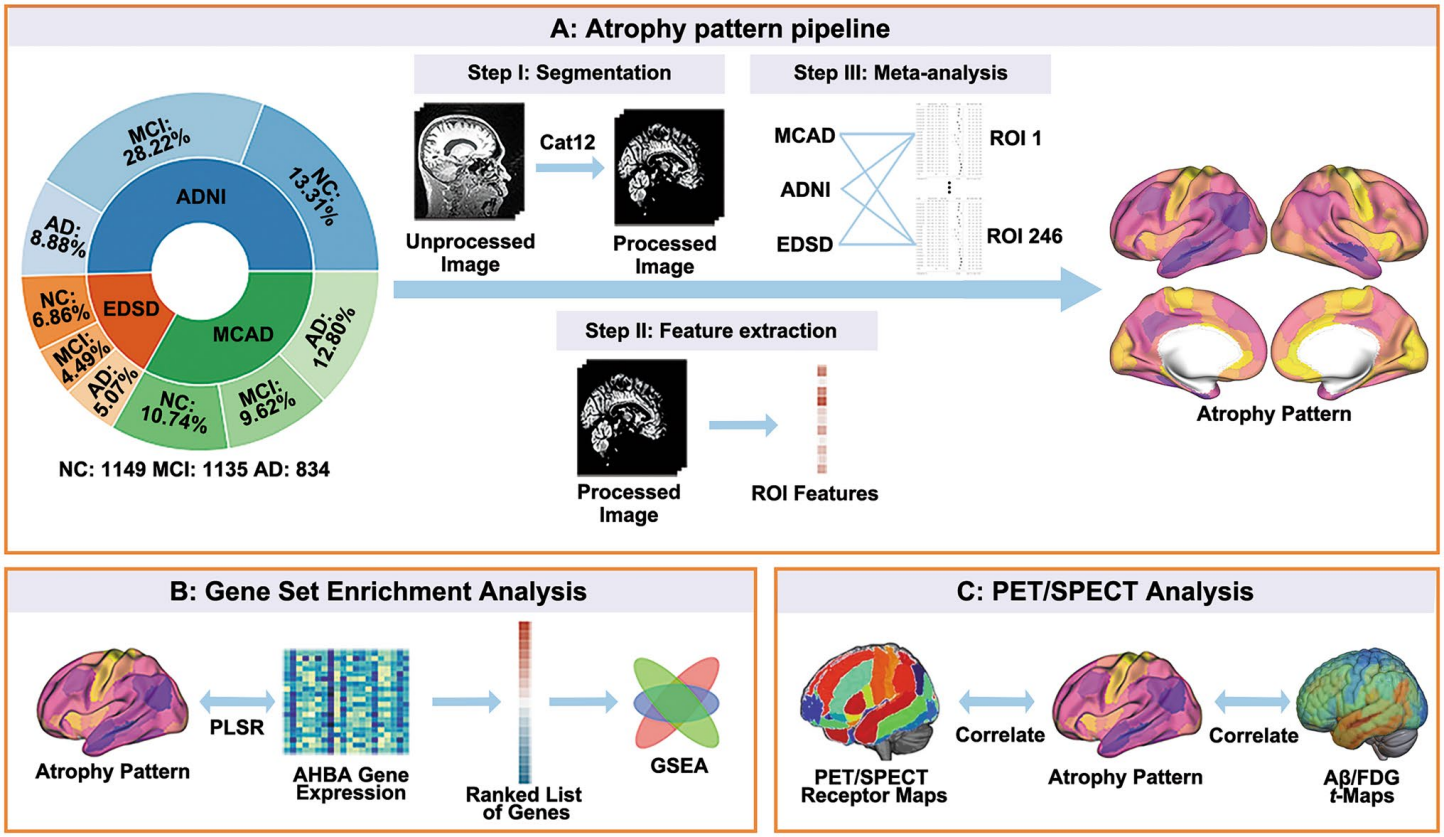
Meta-analysis pipeline. **A** Structural magnetic resonance imaging (sMRI) images go through a unified processing pipeline to extract region of interest (ROI) features (gray matter volume, GMV and cortical thickness, CT), and a meta-analysis is conducted for each ROI. **B** Combining atrophy pattern with gene spatial expression patterns for gene set enrichment analysis. **C** Correlation analyses between atrophy pattern and positron emission tomography (PET) or single photon emission computed tomography (SPECT) features. ADNI, Alzheimer’s Disease Neuroimaging Initiative; EDSD, European diffusion tensor imaging study on dementia; MCAD, Multi-Center Alzheimer’s Disease Imaging; PLSR, partial least squares regression; AHBA, Allen Human Brain Atlas; GSEA, gene set enrichment analysis; Aβ, Amyloid beta; FDG, ^18^F-fluorodeoxyglucose.

**Fig. 2:**
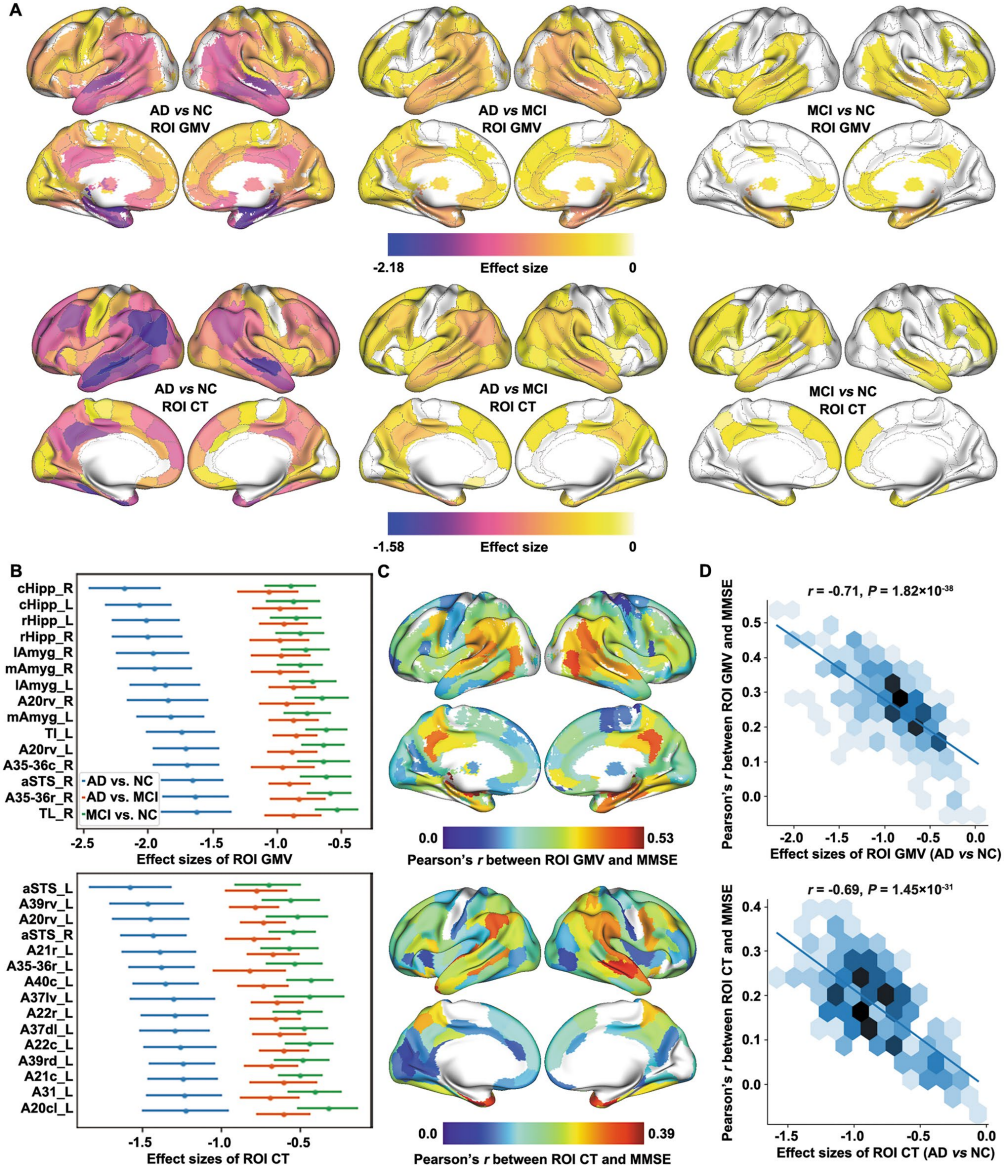
Atrophy patterns based on 23 sites. **A** Gray matter volume (GMV) and cortical thickness (CT) atrophy patterns for Alzheimer’s disease (AD) *vs* normal control (NC), AD *vs* mild cognitive impairment (MCI), and MCI *vs* NC (*P*_FWE_ <0.001). **B** 15 regions of interest (ROI) with the largest absolute effect sizes and their 95% confidence intervals for the ROI GMV/CT meta-analysis. Blue: AD *vs* NC, orange: AD *vs* MCI, green: MCI *vs* NC.** C** Pearson’s *r* between ROI GMV/CT and Mini-Mental State Examination (MMSE) (cognition-related ROI scores).** D** Correlation between ROI atrophy patterns and cognition-related ROI scores.

To understand the widespread impaired gray matter patterns associated with cognitively impaired states, the atrophy pattern of subjects from each group was established as case-control pairs for the meta-analyses (e.g., patterns AD *vs* NC, AD *vs* MCI, and MCI *vs* NC) by comparing the brain structures of AD, MCI, and NC subjects from 23 sites (Fig. 1). For GMV, significant atrophy was found in 235 ROIs in the AD *vs* NC, 190 ROIs in the AD *vs* MCI, and 111 ROIs in the MCI *vs* NC groups (*P*_FWE_ <0.001, Fig. 2A). These results indicate that, even in the MCI stage, specific regions in the brain show notable degeneration, and progressive deterioration of neuronal structures as the disease advances from MCI to AD. The regions showing the most significant atrophy in both MCI and AD stages included the hippocampus (cHipp_R/L and rHipp_R/L), amygdala (IAmyg_R/L and mAmyg_R), and temporal lobe (including aSTS, A20rv, A35/36r, A35/36c, TL and TI) (Fig. 2B). A total of 233 brain region GMVs showed a significant correlation with MMSE (*P*_FWE_ <0.001, Fig. 2C), the ROIs most associated with cognition are the hippocampus and posterior parahippocampal gyrus. There was a significant correlation between this trend and the degree of atrophy in the corresponding brain regions (*r* = −0.71, *P* = 1.82 × 10^−38^, Fig. 2D). In CT, significant cortical atrophy was demonstrated in 196 ROIs in the AD *vs* NC, 157 ROIs in the AD *vs* MCI, and 90 ROIs in the MCI *vs* NC groups (*P*_FWE_ <0.001, Fig. 2A). The most progressive atrophy was found in both temporal lobes (aSTS_L/R). A total of 174 brain region CTs showed a significant correlation with MMSE (*P*_FWE_ <0.001, Fig. 2C); the ROI most associated with cognition is the parahippocampal gyrus (A35/36r).

**Fig. 3:**
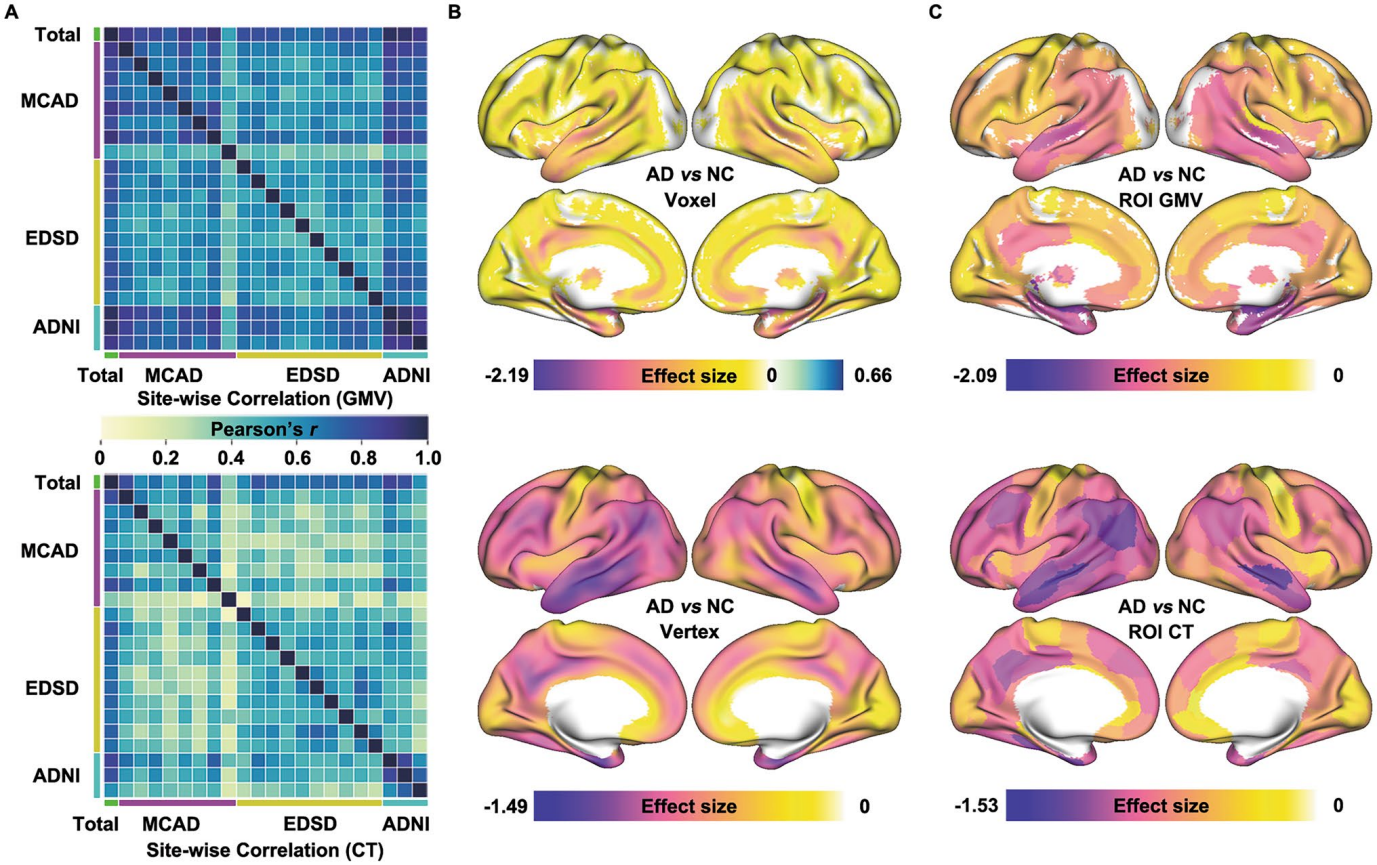
Validation analyses of the meta-analysis.** A** Region of interest (ROI) gray matter volume (GMV) and cortical thickness (CT) effect size correlations between sites in Alzheimer’s disease (AD) *vs* normal control (NC) meta-analysis. **B** Voxel/Vertex-wise meta-analysis result between AD and NC. **C** The meta-analysis results are based on original ROI GMV/CT values without removing covariates such as age, gender, and total intracranial volume. ADNI, Alzheimer’s Disease Neuroimaging Initiative; EDSD, European Diffusion Tensor Imaging Study On Dementia; MCAD, Multi-Center Alzheimer’s Disease Imaging.

To comprehensively evaluate the reliability of our results, we also conducted validation experiments. The site-wise correlation analysis showed a high degree of consistency in the pattern of atrophy across sites (Fig. 3A). Consistent patterns of atrophy were observed in both the voxel/vertex-based meta-analysis (Figs 3B and S4) and the ROI-wise meta-analysis without controlling for covariates (Figs 3C and S5). We applied meta-analyses while regressing the effect of age^2^ (Fig. S6). To further verify the robustness and reproducibility of our findings, we performed additional analyses within each dataset (Figs S7–9), and used the AAL atlas (Fig. S10) and Schaefer 1,000 atlas (Fig. S11) to extract ROI features. Furthermore, we applied the bootstrapping strategy 5,000 times with 80% of the subjects selected for each iteration to calculate the effect size (Fig. S12). These supplementary analyses were highly consistent with the primary results. The analysis results based on neurocombat-harmonized data are also highly consistent with the meta-analysis results (Fig. S13).

### Linking the AD Atrophy Map to Biological Pathways

**Fig. 4:**
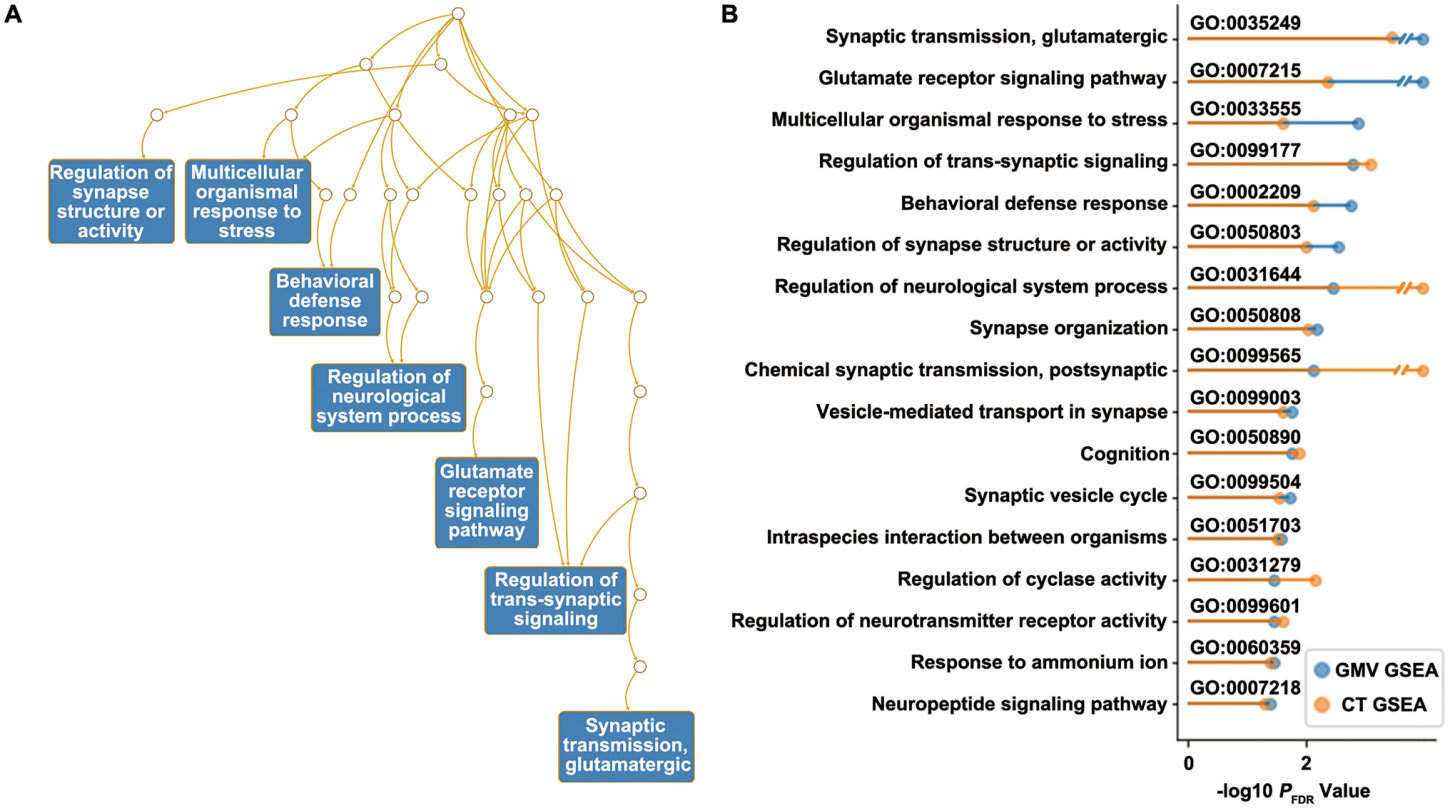
Gene set enrichment analysis (GSEA) results based on Alzheimer’s disease (AD) *vs* normal control (NC) meta-analysis. **A** Directed acyclic graph of the results based on the region of interest (ROI) gray matter volume (GMV) atrophy patterns (*P*_FDR_ <0.005)** B** Significant GSEA results based on the ROI GMV and cortical thickness (CT) atrophy patterns (*P*_FDR_ <0.05).

We applied a PLS-based gene analysis to identify highly correlated genes with robust atrophy patterns in AD. For the AD atrophy assessed using GMV, PLS1 accounted for 42.84% of the total variance (*P* <0.001, 5,000 permutation tests) (Table S2 in Supplementary Material 2). Further, the variance explained by 5,000 SA-preserving surrogate maps was <42.84% (Fig. S14). The most significant gene ontology (GO) terms in GMV-based GSEA are glutamatergic synaptic transmission (GO: 0035249, *P*_FDR_ <10^−4^, *P*_SA_ = 0.004), glutamate receptor signaling pathway (GO: 0007215, *P*_FDR_ = 2.50 × 10^−3^, *P*_SA_ = 0.034), and multicellular organismal response to stress (GO: 0033555, *P*_FDR_ = 1.67 × 10^−3^, *P*_SA_ = 0.002). (Fig. 4A). The enrichment analysis results based on the CT atrophy pattern agreed to a large extent with GMV (Fig. 4B), but there was also a partial discrepancy (Fig. S15). As a result, the enriched GO pathways identified by both GMV and CT analysis can be summarized roughly into three categories: glutamate signal pathway (GO: 0007215, GO: 0035249), cellular stress response (GO: 0033555, GO: 0002209), and synapse structure and function (GO: 0050808, GO: 0050803, GO: 0001578, GO: 0099601, GO: 0099177, GO: 0099003, GO: 0007218, GO: 0099504, GO: 0051932, GO: 0099565) (Fig. S16). The enrichment score for each GO term, GSEA results based on surrogate maps, and *P*_SA_ values can be found in Table S3 in Supplementary Material 3. We also performed tissue and cell-type enrichment analysis (Fig. S17).

**Fig. 5:**
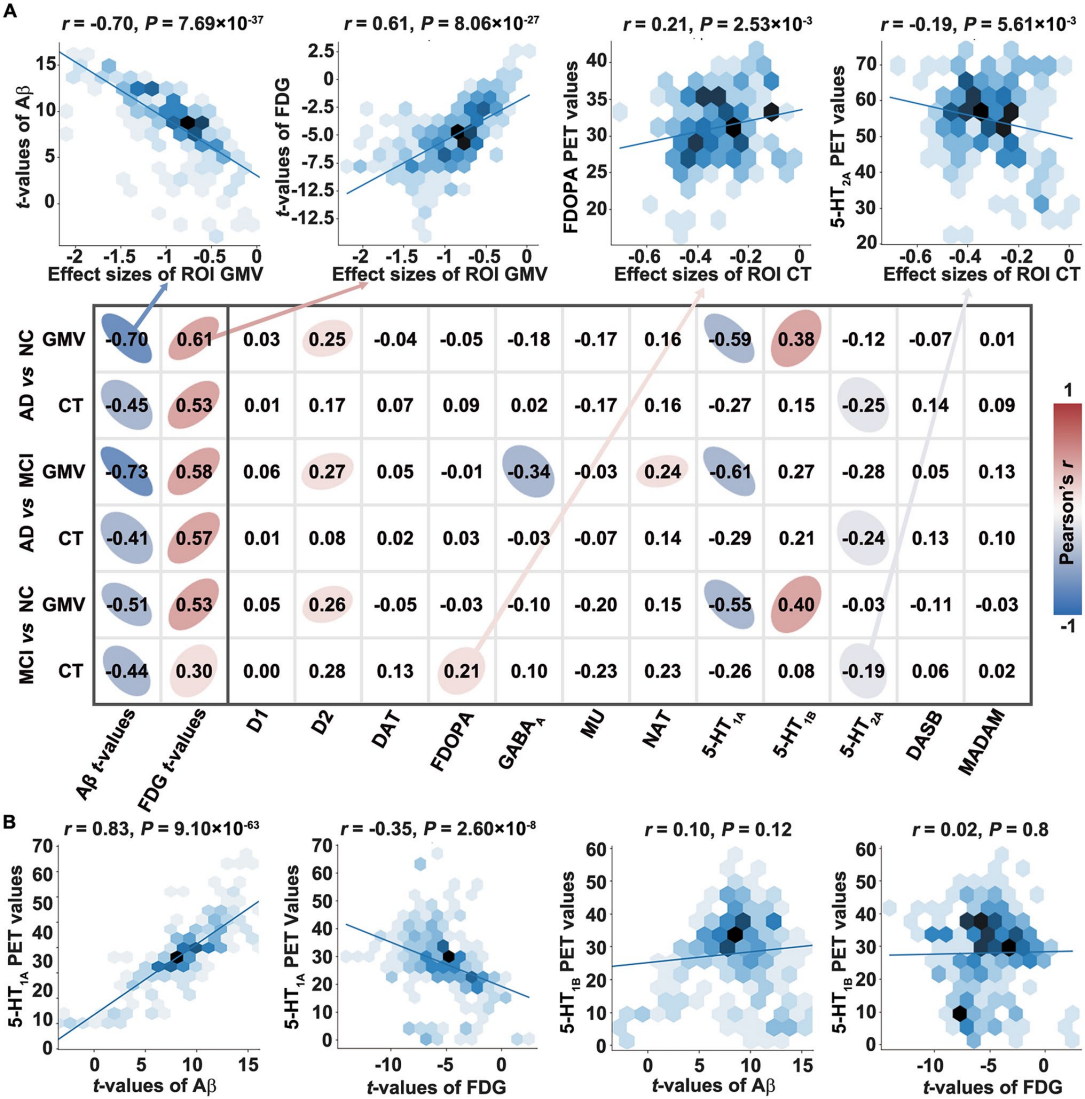
Positron emission tomography (PET) and single photon emission computed tomography (SPECT) analyses results. **A** Overview of the Pearson correlation coefficients between the atrophy (gray matter volume, GMV and cortical thickness, CT) patterns and the Alzheimer’s Disease Neuroimaging Initiative (ADNI) amyloid-beta (Aβ) *t*-values/ADNI ^18^F-fluorodeoxyglucose (FDG) *t*-values/JuSpace neurotransmitter maps (only the values that the *P* and *P*_PET_SA_ are <0.05 at the same time are displayed). **B** Correlation between ROI 5-hydroxytryptamine receptor 1A (5-HT_1A_) or ROI 5-hydroxytryptamine receptor 1B (5-HT_1B_) expression and ROI Aβ/FDG *t*-values. D1, dopamine D1 receptor; D2, dopamine D2 receptor; DAT, dopamine transporter; FDOPA, dopamine synthesis capacity; GABA_A_, gamma aminobutric acid type A receptor; MU, mu opiate receptor; NAT, noradrenaline transporter; 5-HT_2A_, 5-hydroxytryptamine receptor 2A; DASB, serotonin dihydrotetrabenazine tracer; MADAM, ^11^C-*N*,*N*-Dimethyl-2-(2-amino-4-methylphenylthio)benzylamine.

We also assessed AD-related neuropathophysiology using ADNI PET images and evaluated its relationship with atrophy. Integration of the atrophy pattern with ADNI PET images revealed a significant correlation between Aβ deposits and the severity of brain atrophy (*r* = −0.70, *P* = 7.69 × 10^−37^, *P*_Corr_SA_ <0.001, Figs 5A and S18). Concurrently, regions with severe brain atrophy are also accompanied by decreased FDG metabolic activity (*r* = 0.61, *P* = 8.06 × 10^−27^, *P*_Corr_SA_ <0.001). By using the PET-derived and SPECT-derived receptor maps from unrelated healthy subjects, we found that the atrophy pattern was significantly correlated with the expression patterns of 5-hydroxytryptamine (5-HT_1_) receptors, but with different directions (5-HT_1A_: *r* = −0.59, *P* = 3.74 × 10^−24^, *P*_Corr_SA_ <0.001; 5-HT_1B_: *r* = 0.38, *P* = 6.27 × 10^−10^, *P*_Corr_SA_ = 0.016) (Fig. 5A). Though the GABAergic synaptic transmission (GO: 0051932, *P*_FDR_ = 0.034, *P*_SA_ = 0.002) is significantly enriched, only one significant correlation was found between the spatial GABA_A_ receptor expression pattern and atrophy patterns (with AD *vs* MCI GMV, *r* = −0.34, *P* = 1.07 × 10^−9^, *P*_Corr_SA_ = 0.019). We also investigated the crosstalk between the AD PET features and 5-HT_1_ receptors. The result showed that 5-HT_1A_ receptor expression was highly correlated with Aβ deposition (*r* = 0.83, *P* = 9.10 × 10^−63^), while its correlation with FDG change was weaker (*r* = −0.35, *P* = 2.60 × 10^−8^) (Fig. 5B).

## Discussion

This study provided representative brain atrophy patterns in AD through a unified image processing pipeline and ROI-based data-driven meta-analysis of sMRI features with a large sample size (*N* = 3,118). We systematically evaluated the AD-associated alterations in region-specific atrophy, cognition, and brain topographic metabolism patterns. We showed that atrophy in some regions may be more severe and plays a critical role in cognitive decline. Furthermore, we found that the biological pathways associated with brain atrophy are mainly related to the glutamate signal pathway, cellular stress response, and synapse structure and function. These comprehensive findings showed that the cortical volumes in AD patients are smaller in the temporal areas and cortical regions associated with broader memory processing and language processing.

For the AD brain, several literature-based meta-analysis studies have found significant atrophy in the medial temporal lobe (MTL), temporal, and frontal lobes, while atrophy degrees of the parietal and occipital lobes are rarely reported [8, 9]. The literature-based meta-analysis is a powerful tool but suffers several limitations, such as publication bias that may cause the overestimation of effect size and neglect of the negative or null results. A data-driven meta-analysis based on unified processed features is a valuable tool that (a) helps resolve inconsistencies in data origins, (b) avoids the heterogeneity caused by different preprocessing pipelines, (c) provides more precise estimates given a large amount of data from multiple datasets, and (d) provides the ability to compare the degree of atrophy between different brain regions [40].

Benefiting from three multi-site sMRI datasets, we systematically evaluated the robust atrophy patterns for MCI and AD. The regions with the most severe atrophy are distributed in the MTL and limbic system, including the hippocampus, amygdala, and cingulate gyrus. These regions exhibit severe atrophy in the MCI stage and become more severe as the disease progresses. The MTL plays an essential role in memory formation and spatial navigation and is also involved in the consolidation and retrieval of episodic and semantic memory [41]. Specifically, the hippocampus is crucial for forming new memories, and its dysfunction not only causes difficulties in forming new memories but also affects existing memories [42]. Atrophy of the hippocampus is one of the hallmarks of the neurodegenerative changes in AD [43, 44]. The emotion-related limbic system is also one of the regions most affected by atrophy [45, 46]. Amygdala atrophy may cause neuropsychiatric symptoms such as hallucinations, delusions, paranoia, anxiety, and depression, which have also been characterized in AD [47]. In addition to these regions, we also found atrophy in the frontal, parietal, and occipital lobes. Among these, the atrophy of the frontal lobe appeared in the MCI stage and further progressed as the disease developed. In some early studies, frontal lobe atrophy was not found in AD patients [48–50]; meanwhile, some groups also found the frontal lobes are associated with gray matter atrophy [51–54]. These conflicting findings might be due to large variances in previous small sample-size studies. The present data-driven meta-analysis results comprehensively demonstrate that frontal lobe atrophy begins in the early stages of the disease and is widespread in AD. Our study not only substantiates the presence of atrophy in the parietal and occipital lobes but also illuminates the onset of the atrophy that might appear from the MCI stages. These results further support the evidence of atrophy of the MTL in AD but also depict the effect size of the atrophy map of the global brain, giving us a deeper understanding of the degree and progression of atrophy in AD. Here, these findings provide the first quantitative changes of the whole brain and offer potential evidence for better understanding the pathological manifestations of AD.

Furthermore, we investigated the underlying biological mechanisms responsible for the atrophy. The most significantly enriched pathway is related to the glutamate signaling pathway. Glutamate is the major excitatory neurotransmitter in the central nervous system, and its receptor N-methyl-D-aspartate (NMDA) plays a crucial role in learning and memory [55]. Dysfunction of glutamatergic synapses results in a Na^+^ influx, membrane depolarization, and increased intracellular Ca^2+^, promoting membrane depolarization and neuronal excitotoxicity and causing neurodegeneration, which has been well-characterized in AD [55, 56]. A glutamate receptor blocker, memantine, is used clinically to treat moderate to severe AD [57]. Hence, the present study confirmed the association of glutamate with brain atrophy in AD from a data-driven perspective, further emphasizing the importance of glutamate pathway research in slowing AD brain atrophy.

The cellular stress response has also been found to be strongly associated with brain atrophy in AD. It comprises complex cellular processes and molecular mechanisms to restore cellular homeostasis and maintain cell survival. For example, the unfolded protein response is a cellular stress response mechanism activated in response to the accumulation of misfolded proteins, such as Aβ and tau in AD [58, 59]. Cellular stress can also activate the innate immune response and lead to inflammation, a central pathology in AD [60]. GSEA has also revealed a series of pathways related to synapse structure and function, as revealed in previous research [61–63]. Maintaining proper synapse structure and function is essential for normal brain function, and disruption can cause consequences for neural communication and, in some cases, neuron death [64, 65]. These results show some complex mechanisms behind brain atrophy in AD, highlighting the role of the cellular stress response and synapse dysfunction.

Further, the atrophy pattern in AD showed a significant correlation with the Aβ deposition pattern, supporting the possibility that Aβ is involved in the biological processes associated with the atrophy of gray matter. Moreover, our analysis of neurotransmitter expression patterns found significant correlations among serotonin, atrophy, and Aβ deposition patterns. Serotonin receptors are well-known as inhibitory heteroreceptors that regulate the release and activity of glutamate [66, 67]. Loss of glutamatergic pyramidal neurons in the CA1 field of the hippocampus has been found to be relevant to the decrease in 5-HT_1A_ receptor densities [68]. The 5-HT_1A_ receptors are highly concentrated in the cerebral cortex, hippocampus, septum, and amygdala, and they influence the activity of glutamatergic and other neurotransmitters, affecting memory functions [69]. A significant decline in 5-HT_1B_ receptor expression has consistently been seen in post-mortem cortical tissue from AD donors, reflecting the neuronal loss and relevant cognitive decline in this illness [70]. Hence, it is reasonable to hypothesize that these receptors help to preserve functions resulting from brain atrophy in advanced stages of AD. Our findings provide essential insights for thoroughly investigating candidate molecular mechanisms from readily available neuroimaging data, but further mechanism association requires more experiments.

The present study still has some limitations. First, we mainly conducted observational studies based on cross-sectional images, so we need more longitudinal data to corroborate our results. Third, we need further physiological experiments on glutamate and serotonin receptors to verify their relationship with atrophy. Last, since the publicly accessible AHBA gene expression atlas and JuSpace neurotransmitter maps were collected from healthy people, it is necessary to collect AD patients’ data to obtain a more in-depth biological basis for brain atrophy in AD and MCI.

Collectively, this study has successfully identified the robust atrophy patterns in AD using a unified image processing pipeline and data-driven meta-analysis based on sMRI features of a large sample. The analysis showed that the brain atrophy first appears in the MTL, limbic system, and parts of the frontal lobe and spreads to the whole brain, with the most severe atrophy in the hippocampus and amygdala. The glutamate signaling pathway, cellular stress response, and synapse structure and function are strongly associated with atrophy. This study also revealed significant correlations among the serotonin, atrophy, and Aβ deposition patterns. Overall, these findings provide essential insights for developing early detection and treatment strategies for AD.

### Supplementary Information

Below is the link to the electronic supplementary material.

Supplementary file1 (PDF 4874 kb)

Supplementary file2 (XLSX 463 kb)

Supplementary file3 (XLSX 70 kb)

## Data Availability

We can help to run the scripts and share the results maps with requests. The public dataset ADNI supporting the conclusions of this article is available in the ADNI repository, https://adni.loni.usc.edu/. The public dataset EDSD supporting the conclusions of this article is available in the EDSD repository, https://www.neugrid2.eu/index.php/data-portfolio/. The majority of the computations were performed using the Python engine. All the code and statistical imaging data are available at https://github.com/YongLiuLab. Additional datasets supporting the conclusions of this article are included within the article (Figs 1–5) and other files (Supplementary Material 1.docx, Supplementary Material 2.xlsx, and Supplementary Material 3.xlsx).
