# Supplementary material for: Convergent Neuroimaging and Molecular Signatures in Mild Cognitive Impairment and Alzheimer’s Disease: A Data-Driven Meta-Analysis with N = 3,118
Source: Neurosci Bull. 2024 Jun 1;40(9):1274–86. doi: 10.1007/s12264-024-01218-x (PMC11365916; doi:10.1007/s12264-024-01218-x)
Supplement: Supplementary file 1 — Supplementary file1 (PDF 4874 kb) [file 12264_2024_1218_MOESM1_ESM.pdf]

**Supplementary Materials**

## Contents

**Part 1. Description of Database ........ 2**

PL_G, PL_S, and PL_S2 ........ 2
HH_Z ........ 4
QL_W ........ 4
XW_H ........ 5
XW_Z ........ 6
ZJ_L ........ 8
Publicly Available Datasets ........ 8
Quality of the Included Images ........ 8
Included ADNI PET Images ........ 9

**Part 2. Supplementary Meta-analysis ........ 13**

**Part 3. *t*-test Based on Harmonized Data ........ 23**

**Part 4. Gene Set Enrichment Analysis ........ 24**

**Part 5. Tissue and Cell-Type Enrichment Analysis ........ 26**

**Part 6. Relationship Between Gray Matter Features and PET Features ........ 27**

**References ........ 28**

## Part 1. Description of Database

The Multi-Center Alzheimer Disease Imaging Consortium Dataset (MCADI) is a multi-center cohort from different data sites around China. Part of the data from this dataset was used in our previous studies to evaluate generalizable, reproducible, and neuroscientifically interpretable imaging biomarkers in Alzheimer's disease (AD)[1]. This data usage extended our previous studies and included new sites from the PLA hospital (PL_S2) and Zhejiang Provincial People's Hospital (ZJ_L) using the same protocols.

Detailed information, including the ethical permissions and inclusion criteria, can be found elsewhere in our previous studies and supplemental material. Here, with permission, we have rewritten the information to maintain the integrity of the present study (Tables S1 and S2).

### PL_G, PL_S, and PL_S2

This study was approved by the Medical Ethics Committee of the PLA General Hospital, Beijing, China. Written informed consent was given by each enrolled subject or his/her authorized guardian. All participants were recruited by an advertisement (Chinese version). Written consent forms were given by all subjects or their legal guardians under protocols approved by the ethics committee of Chinese PLA General Hospital. Before selection for this study, all the participants were given free physical, psychological, and laboratory examinations, and all patients received professional suggestions for further treatment.

All of the subjects were right-handed and underwent a battery of neuropsychological tests, including the Mini-Mental State Examination (MMSE), Auditory Verbal Learning Test (AVLT), Geriatric Depression Scale (GDS) [2], Clinical Dementia Rating (CDR) [3] and Activities of Daily Living (ADL) Scale. The AVLT consists of a learning trial in which a list of 10 Chinese double-character words are read and the subject is asked to immediately recall as many items as possible. The procedure was repeated a total of 3 times with a new set of words each time, and the immediate recall score was the average number of accurate recalls over the 3 times. After a 5-min delay, each subject was asked to recall the words from the initial list (AVLT-delayed recall). The subjects were then told to identify the 10 studied words, which were intermixed with 10 new words (AVLT recognition).

The recruited AD patients fulfilled the following inclusion criteria: (1) diagnosed using the criteria of the National Institute of Neurological and Communicative Disorders and Stroke and the Alzheimer’s Disease and Related Disorders Association for probable AD; (2) CDR = 1 or 2; (3) currently receiving no nootropic drugs, such as cholinesterase inhibitors; and (4) able to perform the neuropsychological tests and tolerate magnetic resonance (MR) scanning.

The diagnostic criteria for mild cognitive impairment (MCI) were determined as previously described [4] and included the following: (1) memory complaints lasting at least 6 months; (2) CDR = 0.5; (3) intact functional status and ADL <26; and (4) lack of dementia. The criteria for the NCs included the following: (1) normal physical status; (2) CDR = 0; and (3) no memory complaints.

The exclusion criteria for the participants in this study included the following: (1) metabolic conditions, such as hypothyroidism and vitamin B12/folic acid deficiencies; (2) psychiatric disorders, such as schizophrenia and depression; (3) infarction or brain hemorrhaging, as indicated by MR/computed tomography (CT) imaging; and (4) Parkinsonian syndrome, epilepsy, or other nervous system diseases that can influence cognitive function. In addition, patients with a metallic foreign body, such as a cochlear implant, heart stent, or other relevant MR scanning contraindications, were excluded from the study.

MR images for PL_S were acquired on a 3.0 T MR scanner (Siemens Skyra, Siemens, Germany). T1-weighted MR images were acquired using a magnetization-prepared rapid acquisition gradient echo (MP-RAGE) sequence (repetition time (TR) = 2530 ms; echo time (TE) = 3.43 ms; flip angle (FA) = 7°; field of view (FOV) = 256 mm × 256 mm; matrix = 256 × 256; inversion time = 1100 ms; slice thickness = 1 mm, no gap; 192 slices).

MR images for PL_G were acquired on a 3.0 T MR scanner (GE Signa HDx, GE, USA). T1-FSPGR anatomical scans were acquired with the following parameters: TR = 7 ms; TE = 2.9 ms; FA = 8°; FOV = 240 mm × 240 mm; matrix = 256 × 256; inversion time = 450 ms; slice thickness = 1.2 mm, no gap; 166 slices.

Further details are available in previously published studies [5–15].

**HH_Z**

This dataset followed the protocol used in PL_G and PL_S and was approved by the Medical Ethics Committee of Tianjin Huanhu Hospital, Tianjin, China. The patients were recruited from the memory clinic of the neurology department of Tianjin Huanhu Hospital. The control subjects were recruited from the local community using advertisements. Written informed consent was given by each enrolled subject or his/her authorized guardian. The participants underwent general physical, psychological, and laboratory examinations prior to enrollment in the formal study. The participants did not take medications that might have influenced their cognition during the scans, and all patients received professional suggestions for further treatment.

MR images were acquired on a 3.0 T MR scanner (Magnetom Trio, Siemens, Germany). T1-weighted MR images were acquired using an MP-RAGE sequence (TR = 2000 ms; TE = 2.3 ms; FA = 9°; FOV = 232 mm × 256 mm; matrix = 232 × 256; inversion time = 900 ms; slice thickness = 1 mm, no gap; 192 slices; one subject with FOV = 222 mm × 208 mm and matrix = 256 × 240; one subject with FOV = 216 mm × 256 mm and matrix = 216 × 256).

**QL_W**

This dataset followed the protocol used in PL_G and PL_S and was approved by the Medical Ethics Committee of Qilu Hospital of Shandong University, Jinan, China. The patients were recruited from the memory clinic of the Department of Neurology and Radiology, Qilu Hospital of Shandong University. The control subjects were recruited from the local community using advertisements. Written informed consent was given by each enrolled subject or his/her authorized guardian. The participants underwent general physical, psychological, and laboratory examinations prior to enrollment in the formal study. The participants did not take medications that might have influenced their cognition during the scans, and all patients received professional suggestions for further treatment.

MR images were acquired on a 3.0 T MR scanner (Siemens Verio, Siemens, Germany). T1-weighted MR images were acquired using an MP-RAGE sequence (TR = 1900 or 2000 ms; TE = 2.3 ms; FA = 9°; FOV = 256 mm × 256 mm; matrix = 256 × 256; inversion time = 900 ms; slice thickness = 1 mm, no gap; 176 slices).

Related publications can be found elsewhere [16].

**XW_H**

The study was approved by the Medical Research Ethics Committee and Institutional Review Board of Xuanwu Hospital, Beijing, China (ClinicalTrials.gov identifiers: NCT02353884 and NCT02225964). Some of the data have been used in previous studies, and detailed information can be found elsewhere [17,18].

All subjects underwent a series of standardized clinical evaluations, including a medical history interview, a neurological examination, and a battery of neuropsychological tests. The neuropsychological tests included the Chinese version of the MMSE, the Beijing version of the Montreal Cognitive Assessment (MoCA) [19], the CDR Scale [3], the AVLT [20], an ADL assessment, the Hachinski Ischemic Scale, the Hamilton Depression Rating (HAMD) Scale [21], and the Center for Epidemiologic Studies Depression scale [22]. Confirmation of diagnosis for all subjects was made by the consensus of at least two experienced neurologists at the Neurology Department of Xuanwu Hospital. The diagnoses were based on the data available from the neuropsychological assessment evaluation, a battery of general neurological examinations, and subject symptoms as well as functional capacity reports.

The inclusion criteria for an MCI diagnosis included the following [23]: (a) memory complaints confirmed by an informant; (b) objectively impaired memory confirmed by neuropsychological tests; (c) a definitive history of cognitive decline; (d) not meeting the criteria for dementia according to the Diagnostic and Statistical Manual of Mental Disorders, Fourth Edition, Revised (DSM-IV-R); and (e) a CDR score of 0.5.

AD subjects were diagnosed according to the National Institute of Aging-Alzheimer's Association (NIA-AA) criteria for clinically probable AD as follows [24,25]: (a) meeting the criteria for dementia; (b) insidious and gradual onset (not sudden) over >6 months; (c) definitive history of declining cognition; (d) initial and most prominent cognitive deficits evident in amnestic or nonamnestic performance; and (e) hippocampal atrophy confirmed by structural magnetic resonance imaging (MRI).

The NC subjects were required to meet the following research criteria: (a) no memory concerns; (b) MMSE and MoCA scores within the normal ranges (adjusted for age, sex, and education); and (c) a CDR score of 0.

The exclusion criteria applied to all subjects included the following: (a) vascular cognitive impairment (Hachinski Ischemic Scale score >4 points) or a history of stroke; (b) severe depression (HAMD score >24 points or Center for Epidemiological Studies Depression Scale score >21 points); (c) other central nervous system diseases that could cause cognitive decline (e.g., epilepsy, brain tumors, Parkinson’s disease, or encephalitis); (d) systemic diseases that could cause cognitive impairments (e.g., anthracemia, syphilis, thyroid dysfunctions, severe anemia, or HIV); (e) history of psychosis or congenital mental growth retardation; (f) severe hypopsia or dysacusis; (g) cognitive decline caused by traumatic brain injury; (h) severe end-stage disease or severe diseases in acute stages; or (i) unable to complete neuropsychological tests or contraindication with MRI.

MR images were acquired on a 3.0 T MR scanner (Magnetom Trio, Siemens, Germany). T1-weighted MR images were acquired using an MP-RAGE sequence (TR = 1900 ms; TE = 2.2 ms; FA = 9°; FOV = 256 mm × 224 mm; matrix = 512 × 448; inversion time = 900 ms; slice thickness = 1 mm, no gap; 176 slices).

**XW_Z**

All participants were recruited by advertisements and supported throughout the testing procedures at a specialized neuropsychological research facility at Xuanwu Hospital in Beijing, China. Patients and informants (usually a family member) were interviewed clinically by a senior psychiatrist (X. Zhang). Written consent forms were given by all subjects or their legal guardians (usually a family member). The study was approved by the ethics committee of Xuanwu Hospital. AD subjects were diagnosed using standard operationalized criteria (DSM-IVR [American Psychiatric Association, 1994] and NINCDS-ADRDA [24]]).

Patients with a diagnosis of AD and a CDR score of 1 were classified as having mild AD, while patients with a CDR score of 2 or 3 were diagnosed with severe AD

MCI was diagnosed according to standard criteria [4,26,27], including subjective memory loss with objective evidence of memory impairment in the context of normal or near-normal performance on other domains of cognitive functioning, minimal impairment of daily living activities, and a CDR score of 0.5. Normal volunteers had a CDR score of 0.

All participants satisfied the following inclusion criteria: (1) no history of an affective disorder within one month prior to assessment; (2) normal vision and audition; (3) able to cooperate with cognitive testing; (4) aged between 50 and 90 years; (5) no clinical history of stroke or other severe cerebrovascular diseases; and (6) no more than one lacunar infarction, without patchy or diffuse leukoaraiosis, on neuroradiological assessment of conventional MR images.

The exclusion criteria included the following: (1) severe general medical disorders of the cardiovascular, endocrine, renal, or hepatic systems; neurological disorders associated with potential cognitive dysfunction, including local brain lesions, traumatic brain injury with loss of consciousness or confusion, and dementia associated with neurosyphilis, Parkinsonism, or Lewy body disease; psychiatric disorders, including depression, alcohol, or drug abuse; (2) concomitant use of psychotropic medication; or (3) insufficient cognitive capacity to understand and cooperate with the study procedures.

All patients underwent a complete physical and neurological examination, an extensive battery of neuropsychological assessments, and standard laboratory tests. In addition, healthy volunteers underwent a brief clinical interview and the MMSE to confirm that they satisfied the exclusion criteria for cognitive deficits, psychoactive drug use, and clinical disorders. Detailed information can be found in our previous studies [28–32].

MR images were acquired on a 3.0 T MR scanner (Magnetom Trio, Siemens, Germany). T1-weighted MR images were acquired using an MP-RAGE sequence (TR = 2000 ms; TE = 2.6 ms; FA = 9°; FOV = 256 mm × 224 mm; matrix = 256 × 224; inversion time = 900 ms; slice thickness = 1 mm, no gap; 176 slices).

**ZJ_L**

The subjects were recruited from the Memory Clinic of Zhejiang Provincial People's Hospital. All participants were right-handed and were asked to provide written informed consent, and the study was approved by the local Ethics Committee of Zhejiang Provincial People's Hospital[33].

MRI data were acquired using a G.E. 3-Tesla scanner (MR 750) equipped with an 8-channel head coil (GE, USA) at Zhejiang Provincial People's Hospital. Three-dimensional T1- magnetization fast spoiled gradient echo (FSPGR) sagittal images were collected using the following parameters: slice thickness/gap = 1/0 mm; in-plane resolution = 256 × 256; TR = 6.6 ms; TE = 2.9 ms; inversion time = 450 ms; flip angle = 90°; FOV = 256 mm × 256 mm; and voxel size = 1 mm × 1 mm × 1 mm, 192 sagittal slices (Note: Among all the subjects, one had 190 slices, one had 188 slices, one had 184 slices, one had 182 slices, one had 168 slices, one had 164 slices, and two had 160 slices).

**Publicly Available Datasets**

ADNI: Additional information about the Alzheimer's Disease Neuroimaging Initiative (ADNI) dataset can be found at http://adni.loni.usc.edu/wp-content/uploads/how_to_apply/ADNI_Acknowledgement_List.pdf. EDSD: For the acquisition protocol of the EDSD dataset, please refer to their paper [34].

**Quality of the Included Images**

First, we performed a visual check (Y.L, X.K) of all the T1-weighted brain images, and then all the images were processed using the same pipeline. All the MRIs were preprocessed with the standard steps of the CAT12 segmentation process to extract grey matter images and surface data [35]. All the sMRI data were bias-corrected, segmented, and registered to MNI space. The Cat12 toolkit provided a quality score for each subject according to image resolution, noise, bias, and IQR (http://www.neuro.uni-jena.de/cat12-html/cat_methods_QA.html). We removed any subject's data whose image quality was <0.6 (a total of 50 subjects were excluded, leaving 3118 subjects for analysis, Fig. S1) after we considered the reliability of the analysis results [36]. Overall image quality statistics reported by the CAT12 segmentation process of the 3118 used subjects are provided in Fig. S2.

## Included ADNI PET Images

For 1003 subjects with Aβ PET images from the ADNI dataset, there are 291 AD subjects (118 female [41%]; 173 male [59%]; MMSE, 21.49 ± 4.37), 379 MCI subjects (150 female [40%]; 229 male [60%]; MMSE, 27.69 ± 2.06), and 333 NC subjects (166 female [50%]; 167 male [50%]; MMSE, 29.10 ± 1.20). The above information is shown in Fig. S3.

For 912 subjects with FDG PET images from the ADNI dataset, there are 258 AD subjects (107 female [41%]; 151 male [59%]; MMSE, 21.33 ± 4.46), 364 MCI subjects (152 female [42%]; 212 male [58%]; MMSE, 27.82 ± 1.89), and 290 NC subjects (144 female [50%]; 146 male [50%]; MMSE, 29.10 ± 1.20).

**Supplementary Table**

**Table S1** MRI scanner and image acquisition protocol information

| Cohort | Field strength | Brand | Number of head coil channels | Protocol name | Repetition time | Echo time | Flip angle | Field of view | Matrix | Slice number/ thickness (no gap) |
|---|---|---|---|---|---|---|---|---|---|---|
| PL_S | 3.0 T | Siemens Skyra | 20 | MP-RAGE | 2530 ms | 3.43 ms | 7° | 256 × 256 | 256 × 256 | 192/1 |
| PL_G | 3.0 T | GE Signa HD × | 8 | FSPGR | 7 ms | 2.9 ms | 8° | 240 × 240 | 256 × 256 | 166/1.2 |
| HH_Z | 3.0 T | Siemens Trio Tim | 20 | MP-RAGE | 2000 ms | 2.3 ms | 9° | 232 × 256 | 232 × 256 | 192/1 |
| QL_W | 3.0 T | Siemens Verio | 8 | MP-RAGE | 2000/1900 ms | 2.3 ms | 9° | 256 × 256 | 256 × 256 | 176/1 |
| XW_H | 3.0 T | Siemens Trio Tim | 12 | MP-RAGE | 1900 ms | 2.6 ms | 9° | 256 × 224 | 512 × 448 | 176/1 |
| XW_Z | 3.0 T | Siemens Trio Tim | 8 | MP-RAGE | 2000 ms | 2.2 ms | 9° | 256 × 225 | 256 × 224 | 176/1 |
| PL_S2 | 3.0 T | Siemens Skyra | 20 | MP-RAGE | 2530 ms | 3.43 ms | 7° | 256 × 256 | 256 × 256 | 192/1 |
| ZJ_L | 3.0 T | GE 750 | 8 | FSPGR | 7 ms | 2.9 ms | 1° | 256 × 256 | 256 × 256 | 192/1 |

## Supplementary Figures

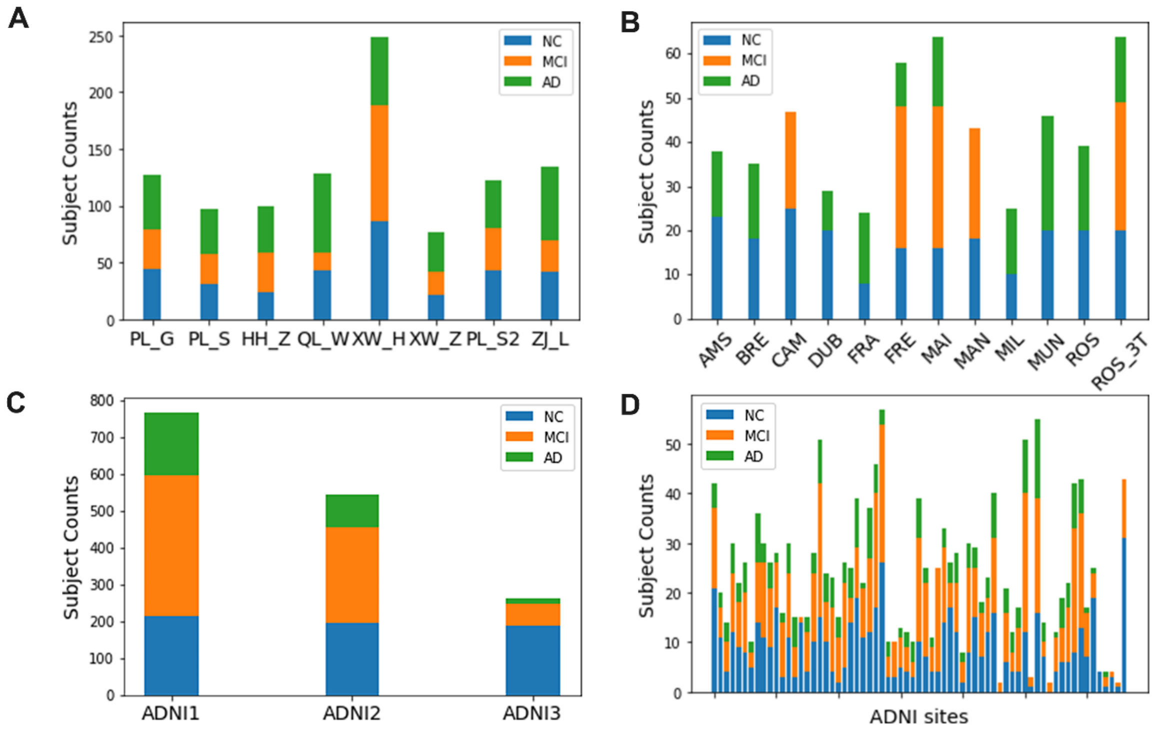


**Fig. S1** Distribution of the number of subjects in three datasets. **A** Subject counts for the in-house (MCAD) dataset. **B** Subject counts for the EDSD dataset. **C** Subject counts for the ADNI dataset sorted by their study phase. **D** Subject counts for the ADNI dataset sorted by their acquisition sites.

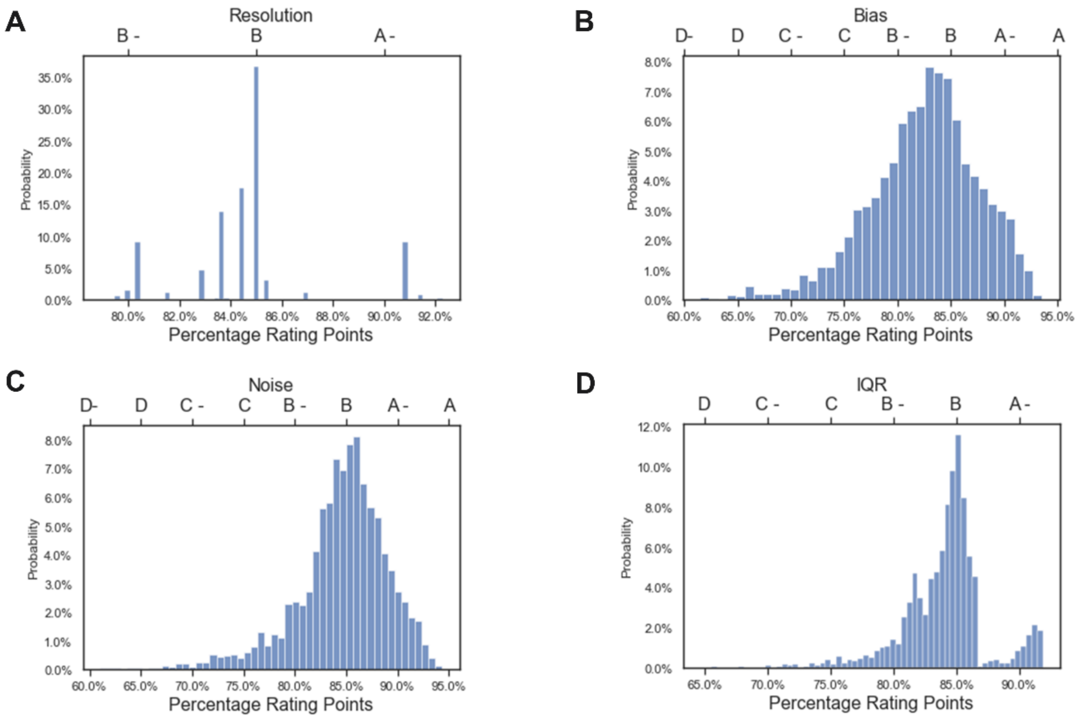


**Fig. S2** The CAT12 segmentation process reports overall image quality statistics. **A** Resolution rating. **B** Bias rating. **C** Noise rating. **D** IQR rating.

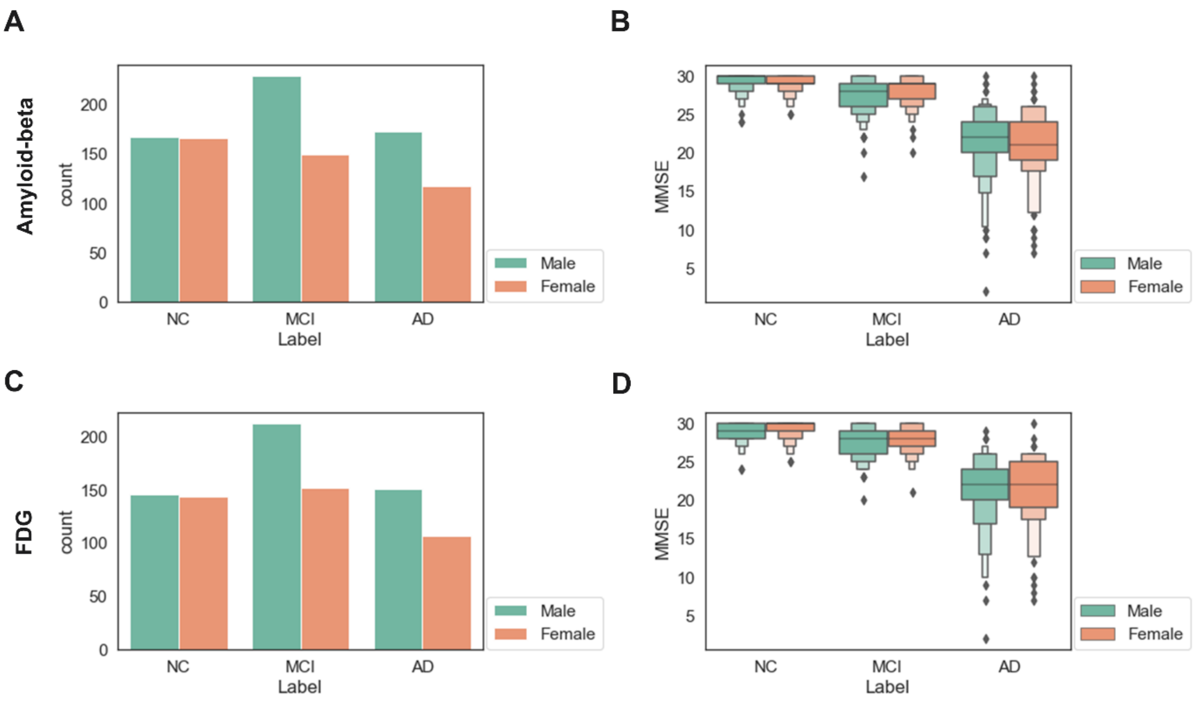


**Fig. S3** Subject counts and MMSE statistics for the Aβ and FDG PET images. **A** Number of subjects with Aβ for each group. **B** MMSE statistics for the subjects with Aβ. **C** Number of subjects with FDG for each group. **D** MMSE statistics for the subjects with FDG.

## Part 2. Supplementary Meta-analysis

In addition to the primary meta-analysis we described in the manuscript, we also performed a series of tests to verify the robustness and reproducibility of our results.

(1) Analysis using voxel/vertex features

We applied meta-analyses using voxel/vertex features that controlled for covariates (Fig. S4).

(2) Analysis using original features

We applied analyses using the original data (without controlling for covariates, Fig. S5).

(3) Analysis with $Age^2$ removed

We applied the analyses using ROI features that controlled for age, $age^2$, gender, and TIV (Fig. S6).

(4) Meta-analysis for each dataset

We conducted meta-analyses that only included subjects in one dataset (Fig. S7–9).

(5) Testing using different brain atlases (AAL atlas, Schaefer 1000 atlas)

We also tested the ROI results using the AAL (116 regions, Fig. S10) and Schaefer 1000 (1000 regions, Fig. S11) atlases. The mean cortical thickness in these atlases was calculated by resampling both the atlas and the individual thickness onto an HCP 32K surface using Cat12.

(6) Random sample analysis

We applied the bootstrapping strategy 5000 times (Fig. S12A). Each time we randomly sampled 80% of the subjects from each cohort. Next, we calculated the effect size and cohort weights using inverse variance with random effect models. Then, we combined all the cohorts' effect sizes to calculate the total effect size. Finally, we compared the effect size of 80% of the subjects with the effect size of all the subjects.

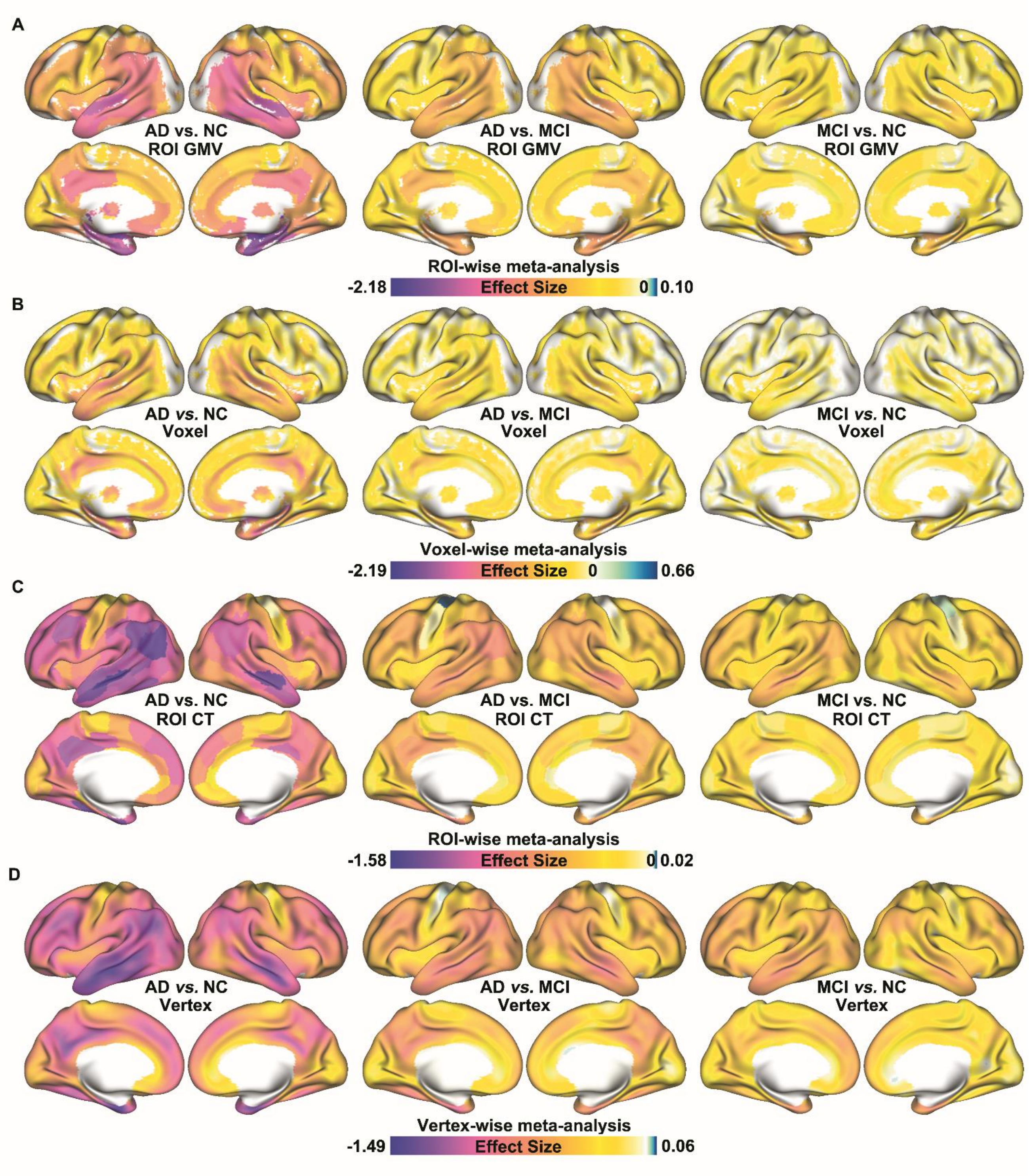


**Fig. S4** Comparison of main ROI-wise results with voxel/vertex results. **A** Main ROI GMV results. **B** Voxel-wise meta-analysis results. **C** Main ROI CT results. **D** Vertex-wise meta-analysis results.

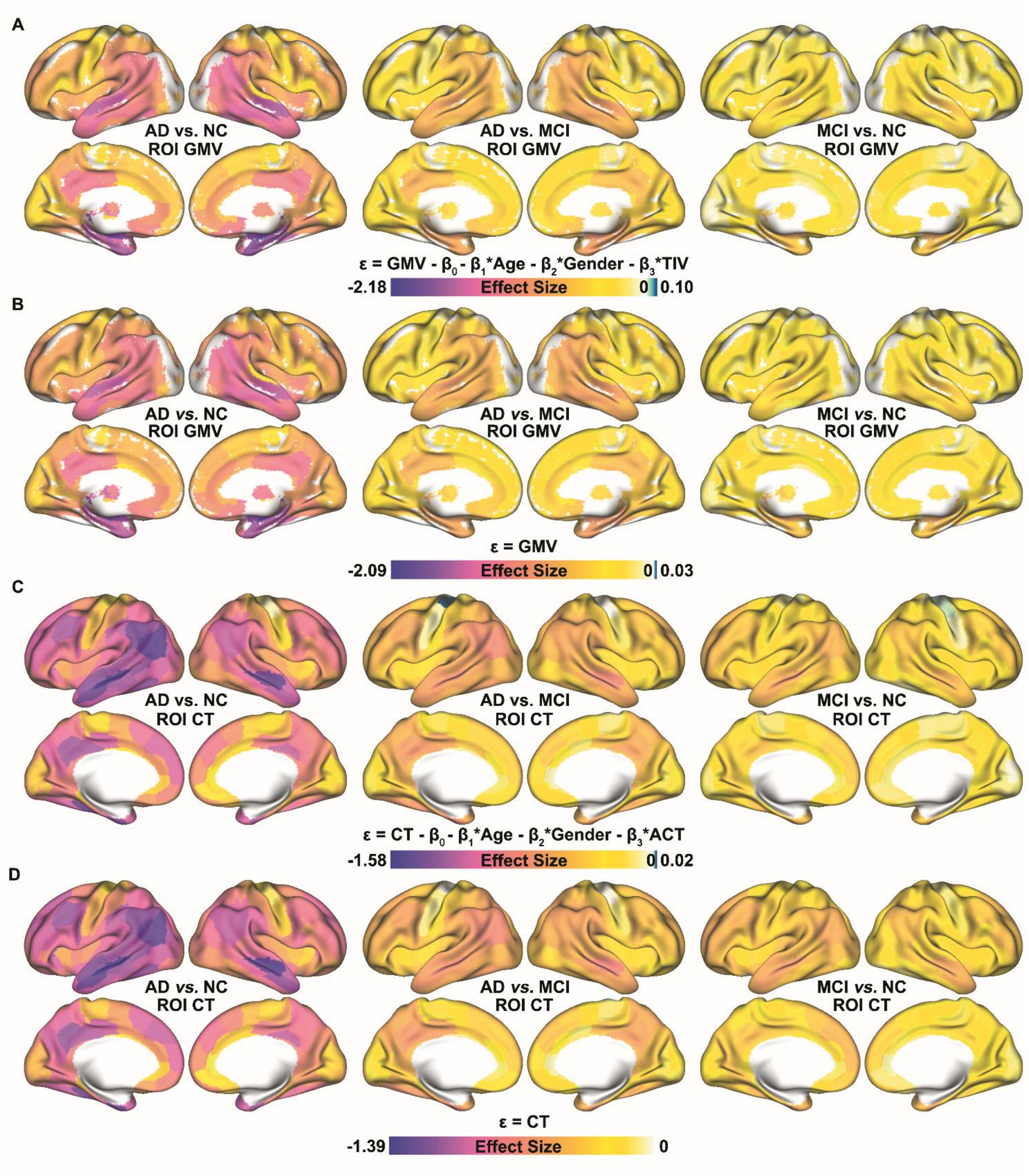


**Fig. S5** Comparison of main results with results without controlling covariates. **A** Main ROI GMV results. **B** ROI GMV meta-analyses results based on original features. **C** Main ROI CT results. **D** ROI CT meta-analyses results based on original features.

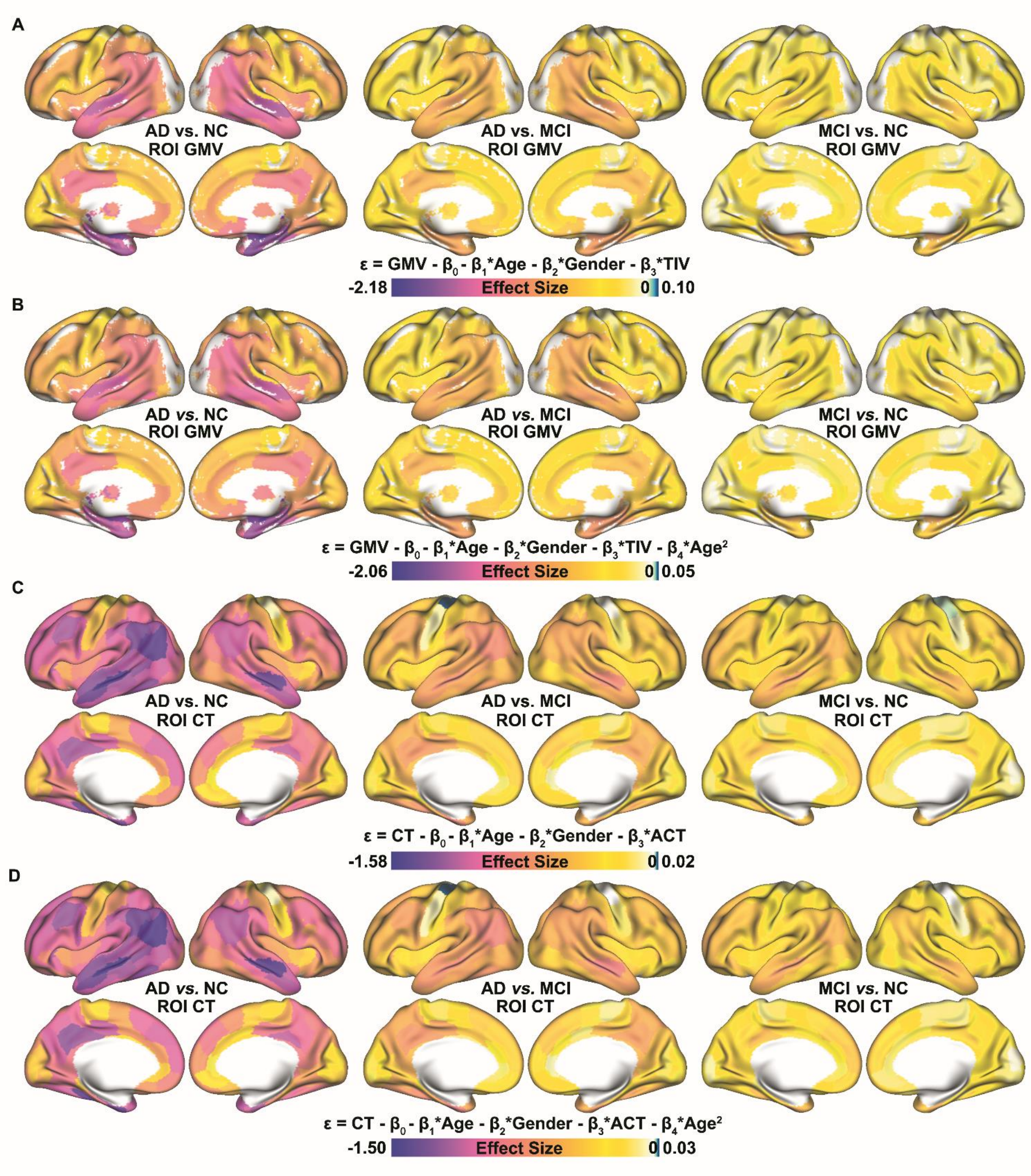


**Fig. S6** Comparison of main results with results controlling age$^2$. **A** Main ROI GMV results. **B** ROI GMV meta-analyses result-based features additionally controlled for age$^2$. **C** Main ROI CT results. **D** ROI CT meta-analyses result-based features additionally controlled for age$^2$.

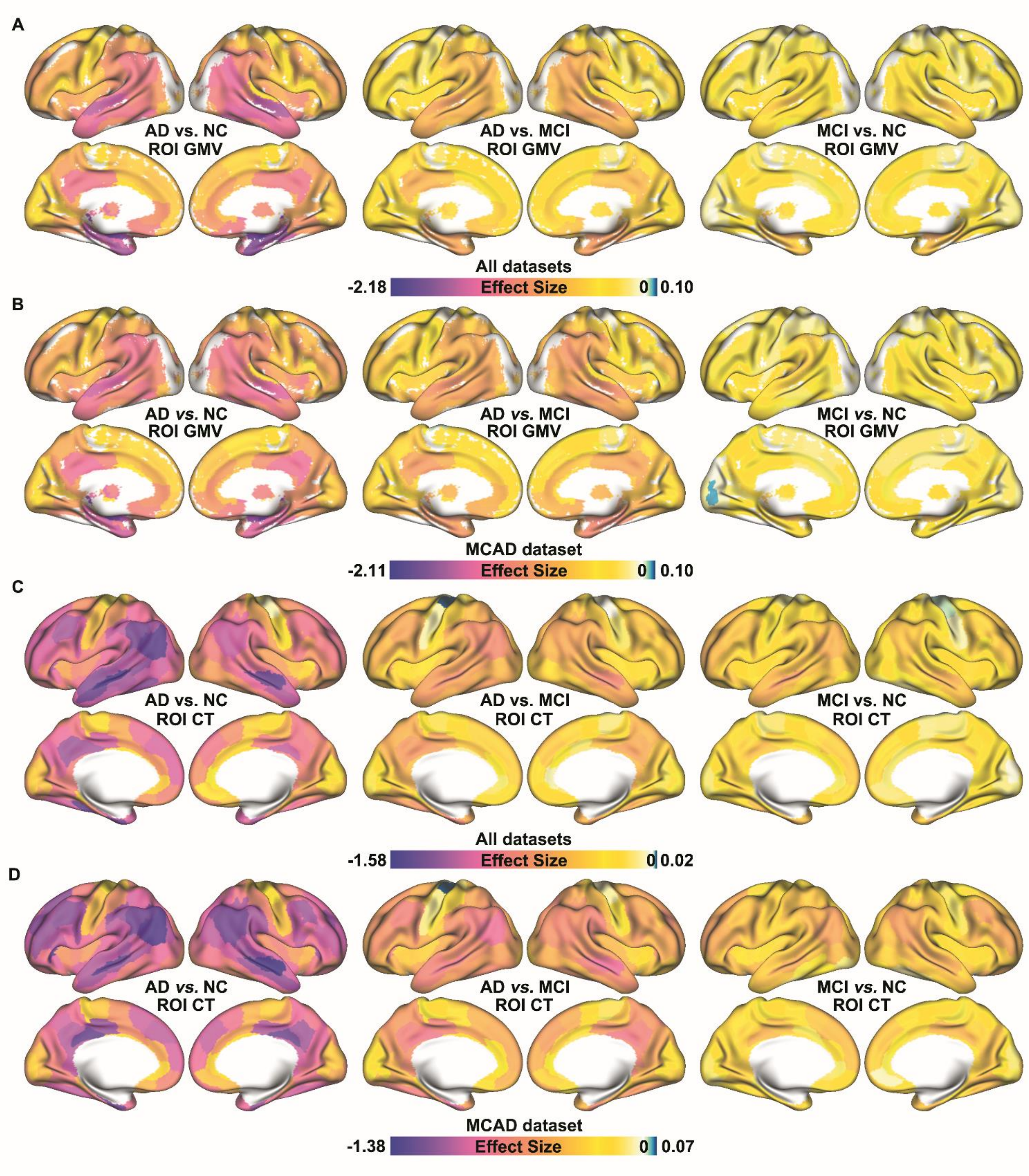


**Fig. S7** Comparison of main results with results based on the MCAD dataset. **A** Main ROI GMV results. **B** ROI GMV meta-analyses results based on the MCAD dataset. **C** Main ROI CT results. **D** ROI CT meta-analyses results based on the MCAD dataset.

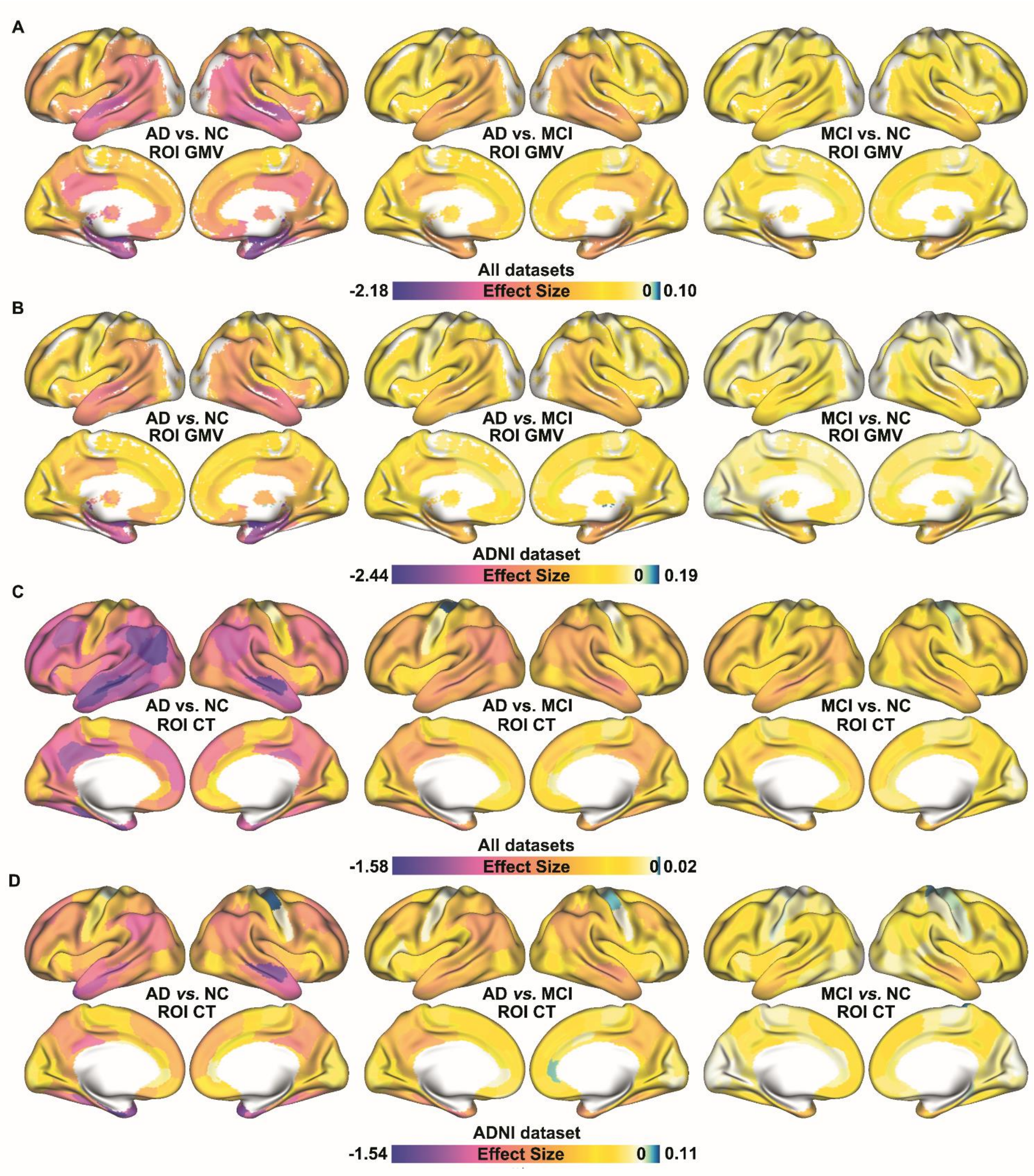


**Fig. S8** Comparison of the main results with results based on the ADNI dataset. **A** Main ROI GMV results. **B** ROI GMV meta-analyses results based on the ADNI dataset. **C** Main ROI CT results. **D** ROI CT meta-analyses results based on the ADNI dataset.

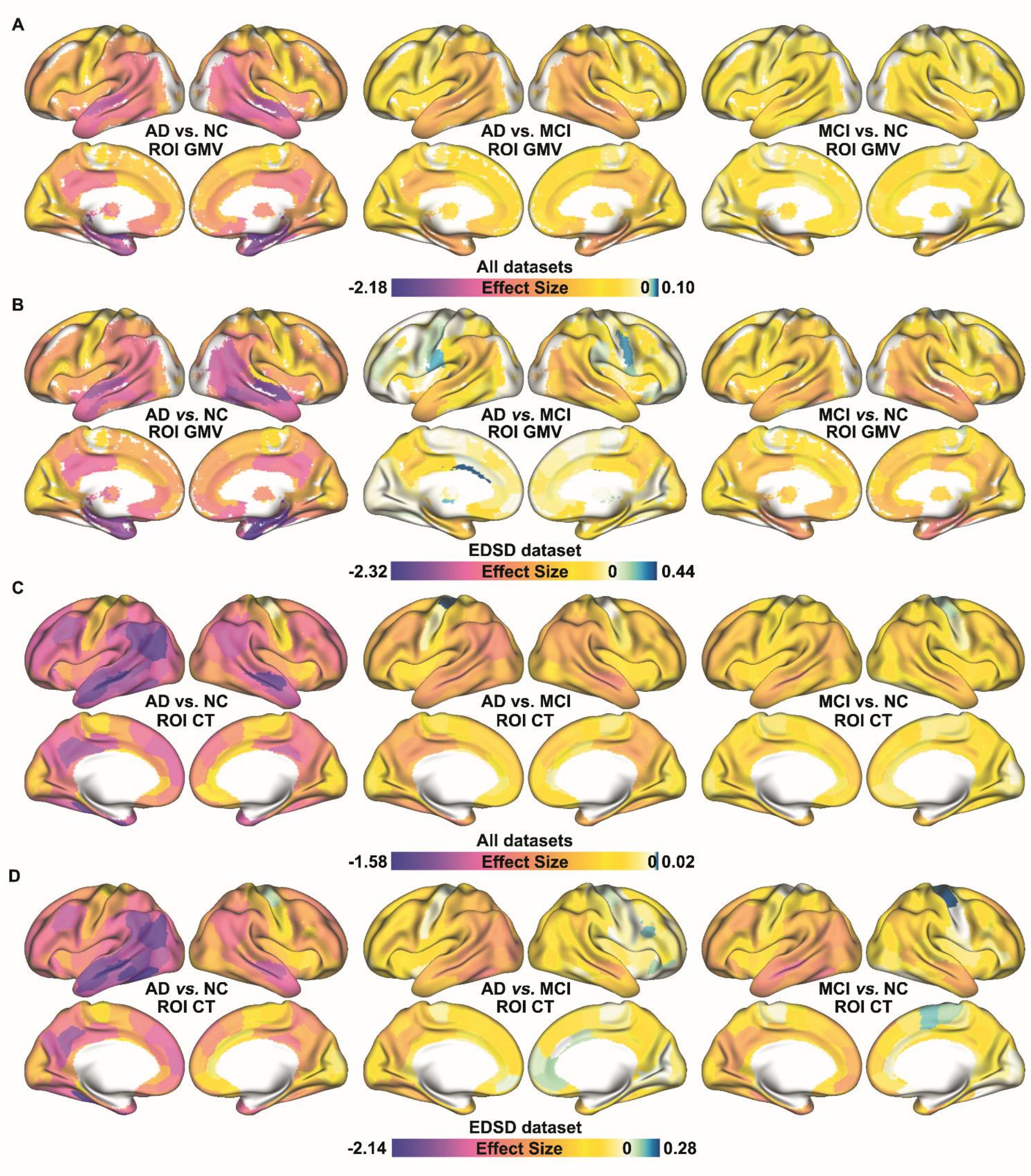


**Fig. S9** Comparison of the main results with results based on the EDSD dataset. **A** Main ROI GMV results. **B** ROI GMV meta-analyses results based on the EDSD dataset. **C** Main ROI CT results. **D** ROI CT meta-analyses results based on the EDSD dataset.

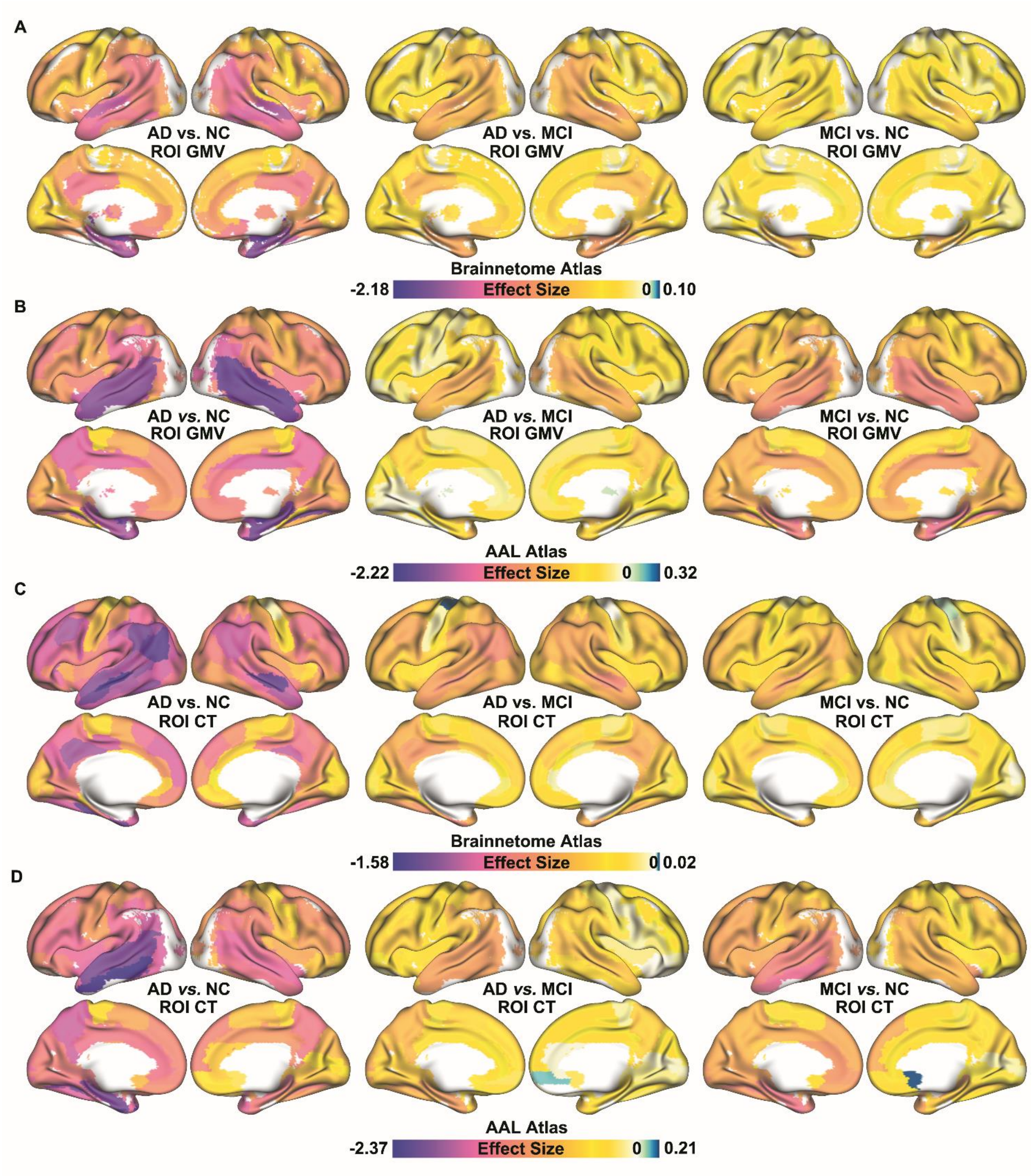


**Fig. S10** Comparison of the main results with results based on the AAL atlas. **A** Main ROI GMV results. **B** ROI GMV meta-analyses results based on the AAL atlas. **C** Main ROI CT results. **D** ROI CT meta-analyses results based on the AAL atlas.

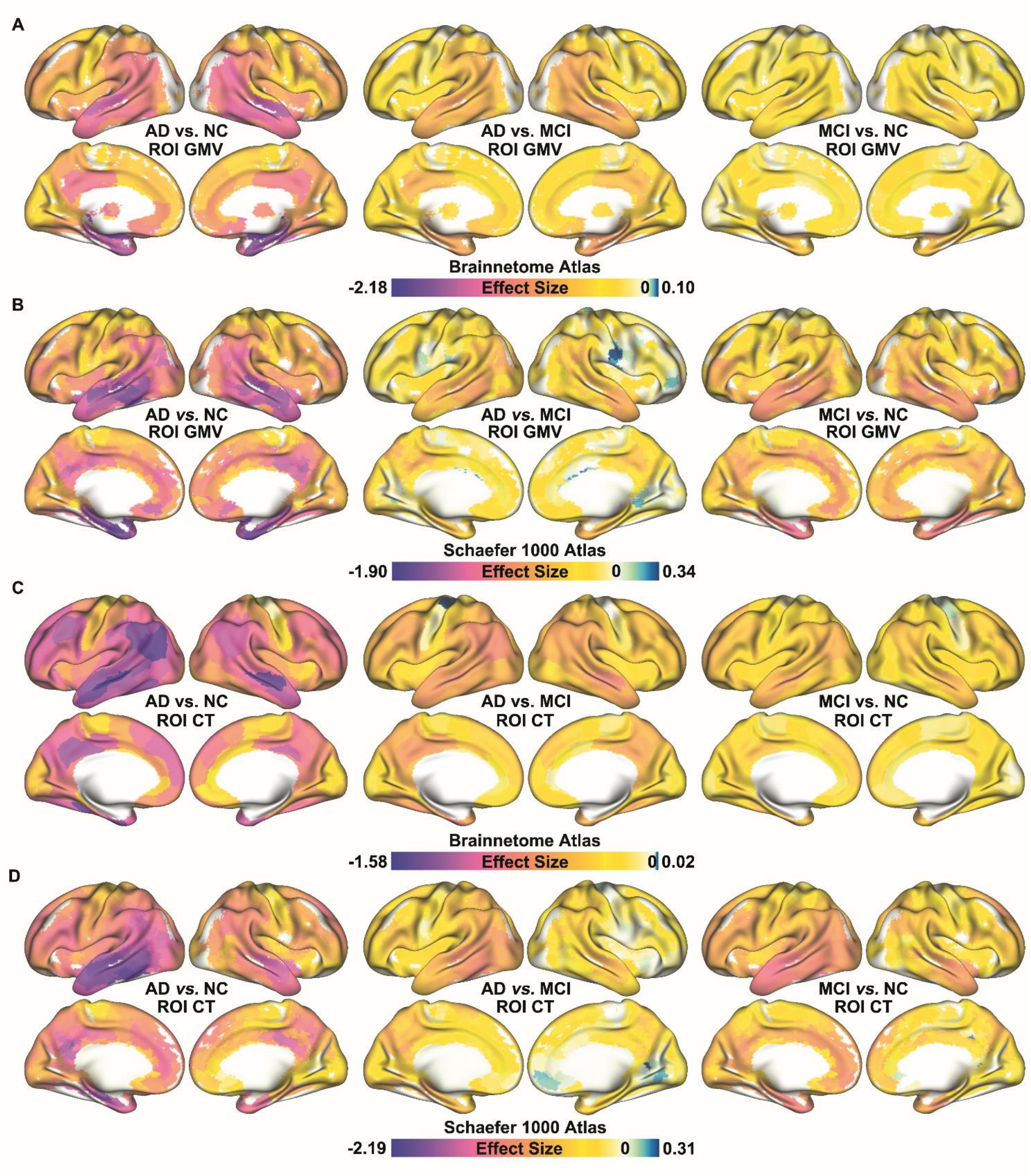


**Fig. S11** Comparison of the main results with results based on the Schaefer atlas. **A** Main ROI GMV results. **B** ROI GMV meta-analyses results based on the Schaefer atlas. **C** Main ROI CT results. **D** ROI CT meta-analyses results based on the Schaefer atlas.

In the process of SIMPLS analysis, we conducted 5000 permutation tests to calculate the *P*-value to improve the reliability of our results (https://github.com/rmarkello/pyls#regression-with-simpls).

We also took 5000 randomly sampled meta-analysis results to perform a PLS analysis and evaluated the correlation between its first component weight and primary PLS analysis (Fig. S12B). PLS was also applied to the single dataset meta results, and their correlation with the primary PLS analysis was $r$ = 0.98 for MCAD, $r$ = 0.99 for EDSD, and $r$ = 0.96 for ADNI.

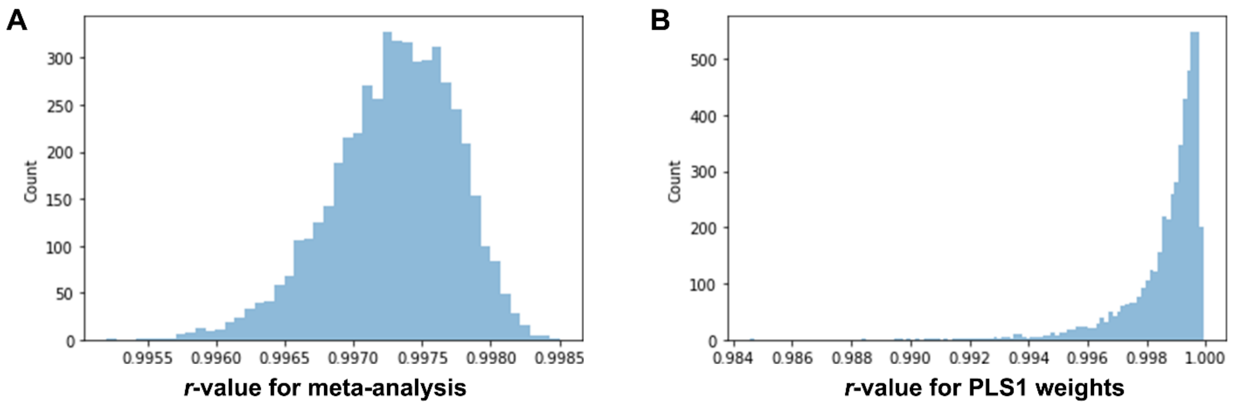


**Fig. S12** Robustness of the meta-analysis and gene PLSR analysis. **A** Random sampling of 80% of the subjects 5000 times was conducted for the meta-analysis, and the *r*-value of the meta-analysis is the correlation coefficient between the meta Cohen's *d* and the main result. **B** The gene PLSR analysis was based on 5000 meta-analysis results, and the *r*-value of the PLS1 weight is the correlation coefficient between the PLS1 weight with 80% of samples and the main PLS1 weight with the entire dataset.

## Part 3. *t*-test Based on Harmonized Data

To further validate and strengthen our results, we used an alternative statistical analysis method on the gray matter data. We first used neuroCombat [37] to harmonize the GMV and CT to reduce the impact of site effects. When performing harmonization, we use the label, age, gender, TIV, or average CT as covariates. Then we applied a *t*-test to evaluate the atrophy based on harmonized data. The results are shown in Fig. S13.

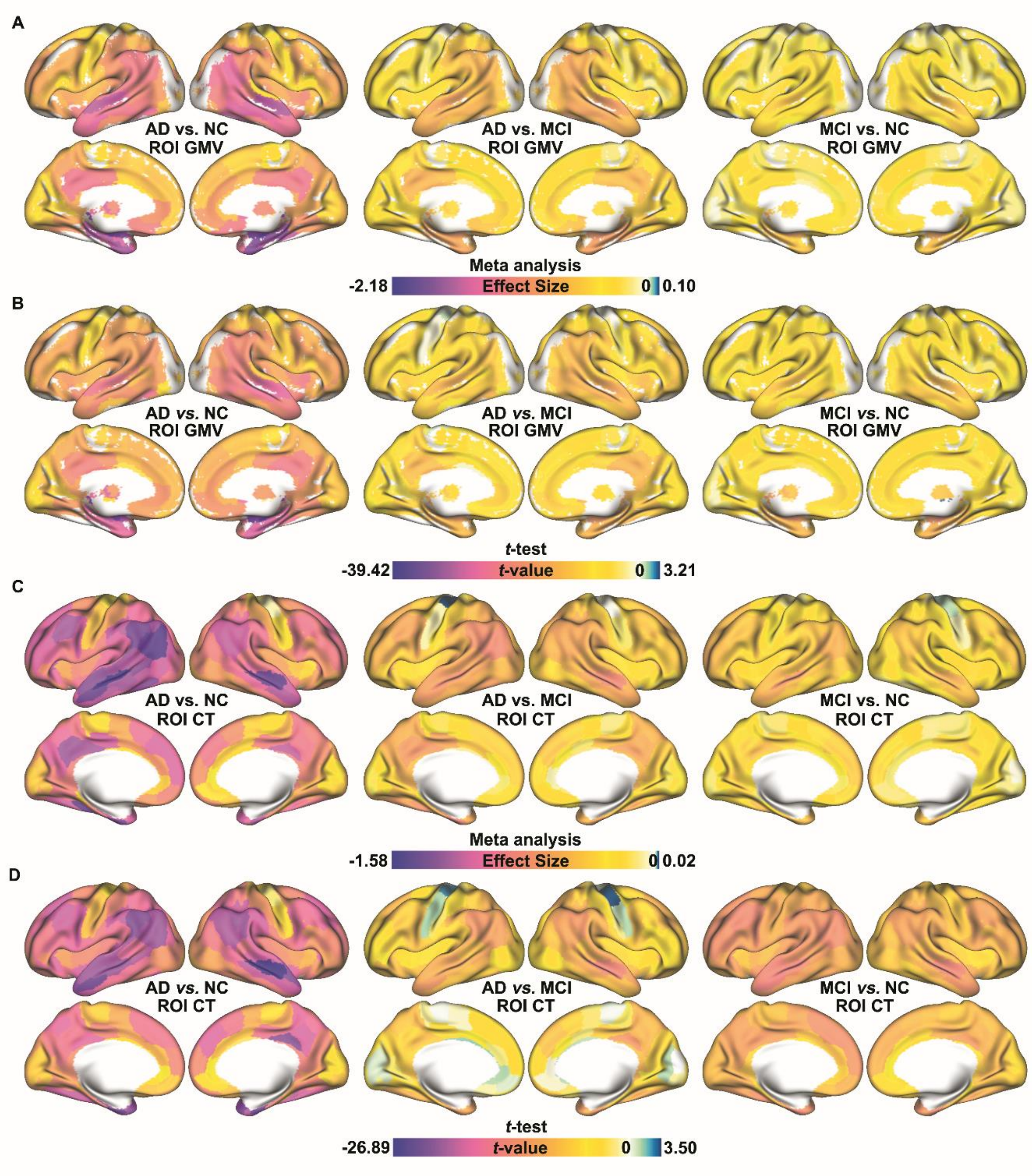


**Fig. S13** Comparison of main meta-analysis results with *t*-test results based on harmonized data. **A** Main ROI GMV results. **B** The *t*-test results based harmonized ROI GMV. **C** Main ROI CT results. **D** The *t*-test results based harmonized ROI CT.

## Part 4. Gene Set Enrichment Analysis

We generate 5000 spatial autocorrelation-preserving surrogate maps using brainSMASH [38,39] to test the significance of our PLSR results (Fig. S14A). The variance explained by 5000 spatial autocorrelation-preserving surrogate maps is <42.84% (Fig. S14B).

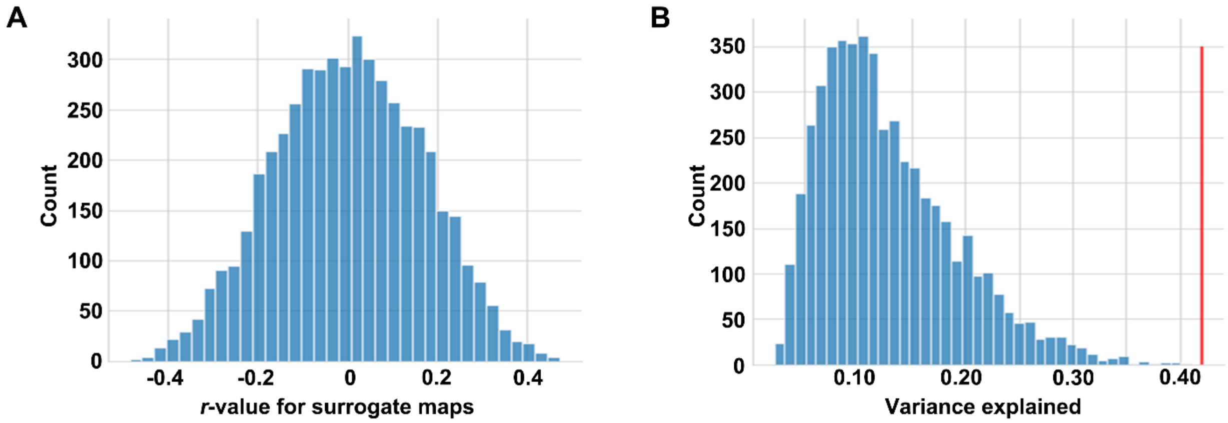


**Fig. S14** Analysis of surrogate maps. **A** Distribution of *r*-values between the surrogate map and the AD *vs* NC ROI GMV effect sizes. **B** Distribution of PLS1 variance explained by PLSR analysis between surrogate map and gene expression, red line indicated the PLS1 variance explained by PLSR analysis between AD *vs* NC ROI GMV effect sizes and gene expression.

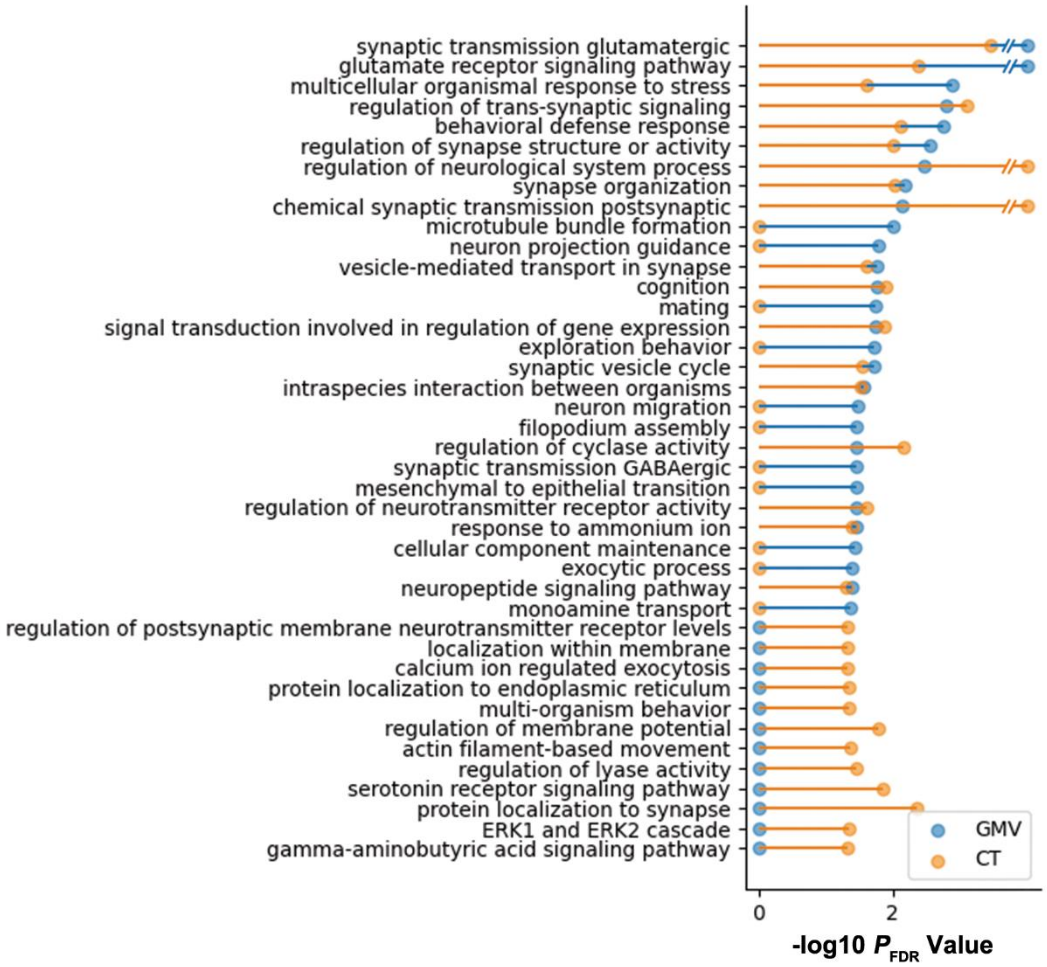


**Fig. S15** A joining of GMV-based GSEA results and CT-based GSEA results.

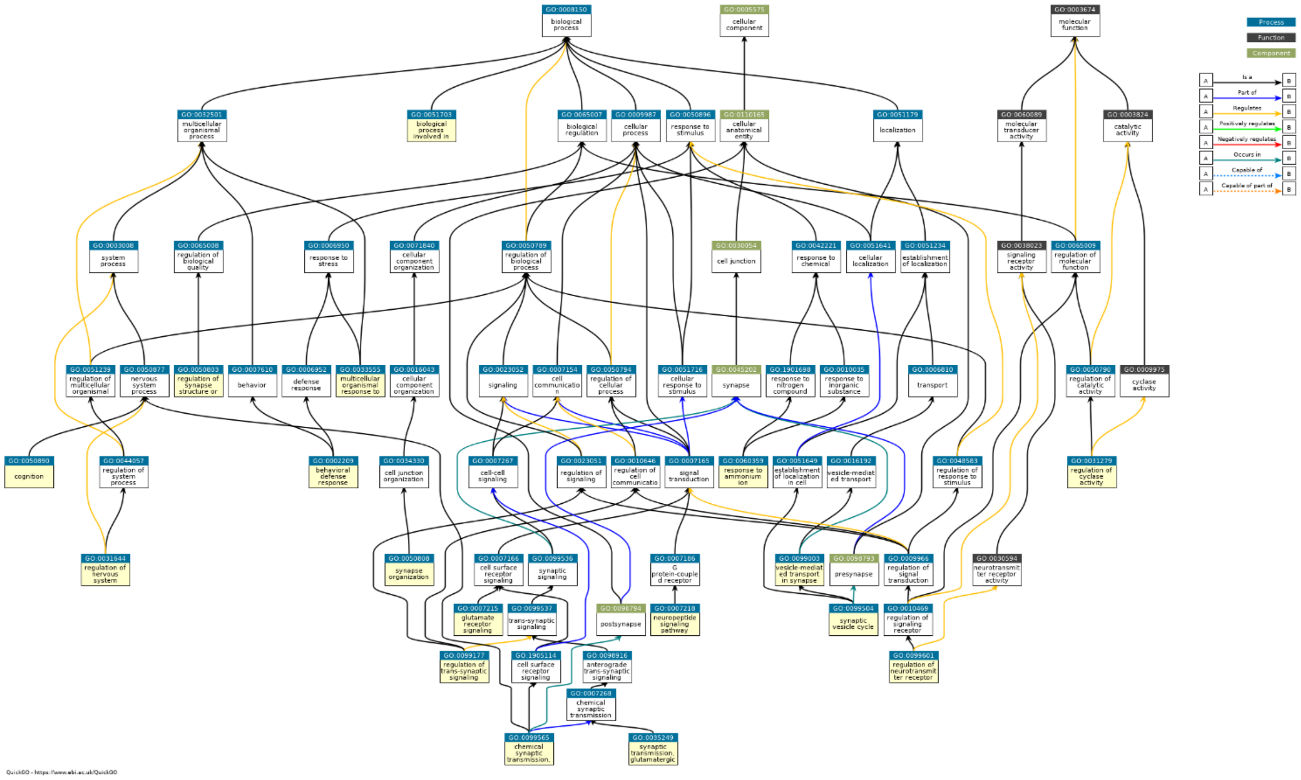


**Fig. S16** A full directed acyclic graph of significant GO terms (enriched in both GMV-based GSEA and CT-based GSEA).

## Part 5. Tissue and Cell-Type Enrichment Analysis

We investigated whether the high-ranking genes associated with AD atrophy patterns are enriched in specific tissues and cell types (Fig. S17).

The 156 genes (1% AHBA genes with the highest weight) from the GMV-based analysis were applied in an enrichment analysis using functional mapping and annotation (FUMA). 152 genes were recognized by the FUMA GENE2FUNC function, and they exhibited significant expression enrichment in brain tissue, especially the hypothalamus ($P_{\mathrm{FDR}} = 9.91 \times 10^{-36}$), anterior cingulate cortex BA24 ($P_{\mathrm{FDR}} = 1.87 \times 10^{-34}$), and amygdala ($P_{\mathrm{FDR}} = 7.74 \times 10^{-34}$).

In the CT-based analysis, the most significant genes were enriched in cortical tissue ($P_{\mathrm{FDR}} = 3.94 \times 10^{-24}$). Further cell-type-specific expression analyses (CSEA) were performed for atrophy-related essential genes and showed that these genes were significantly enriched in Glt25d2 neurons ($P_{\mathrm{FDR}} = 6.57 \times 10^{-7}$) and Ntsr+ neurons ($P_{\mathrm{FDR}} = 2.07 \times 10^{-5}$) within the cortex.

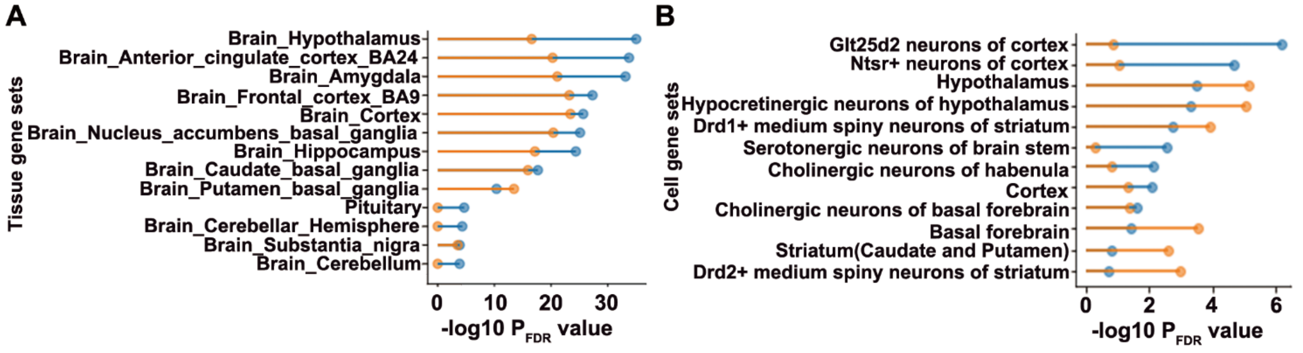


**Fig. S17** Tissue and cell-type enrichment analysis results based on the 1% of the genes with the greatest weights in the PLS result. **A** Significant results of FUMA GENE2FUNC ($P_{FDR}$ <0.001). **B** Significant results of CSEA ($P_{FDR}$ <0.05).

## Part 6. Relationship Between Gray Matter Features and PET Features

We collected subjects with both gray matter images and PET images from the ADNI dataset. We used the Brainnetome atlas to extract the gray matter and PET features of brain regions. And then we applied correlation analysis to each brain region in these subjects, and obtained a correlation value for each region (Fig. S18).

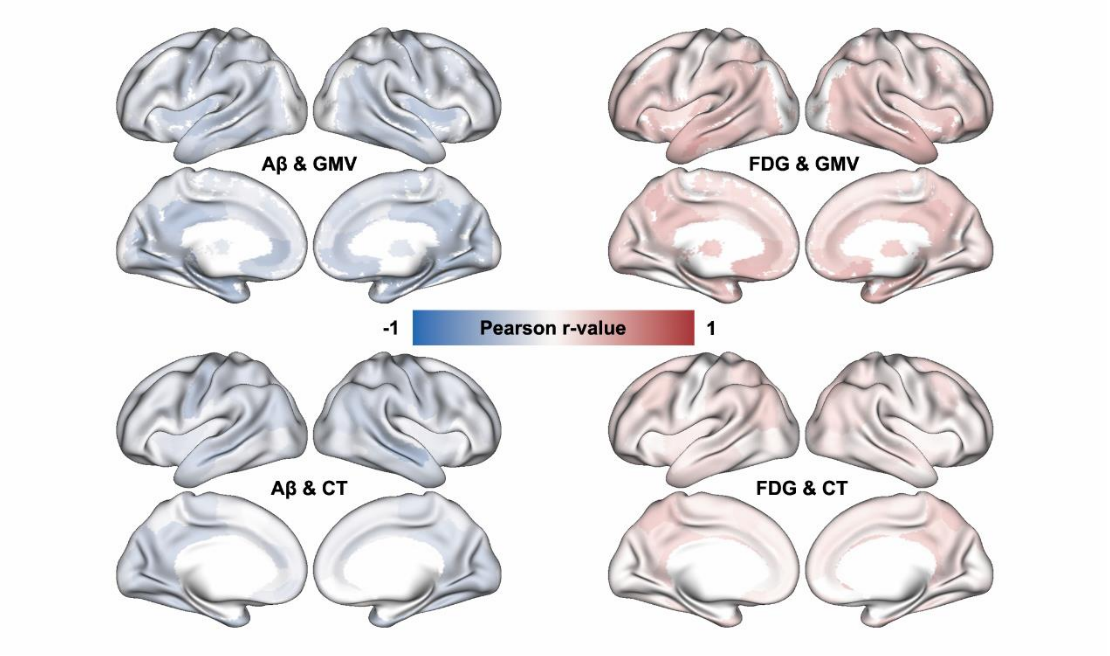


**Fig. S18** ROI-wise correlation between GMV/CT and Aβ/FDG.

## References

[1] Jin D, Zhou B, Han Y, Ren J, Han T, Liu B, *et al*. Generalizable, reproducible, and neuroscientifically interpretable imaging biomarkers for Alzheimer's disease. Adv Sci 2020, 7: 2000675.

[2] Yesavage JA, Brink TL, Rose TL, Lum O, Huang V, Adey M, *et al*. Development and validation of a geriatric depression screening scale: A preliminary report. J Psychiatr Res 1982, 17: 37–49.

[3] Morris JC. The Clinical Dementia Rating (CDR): Current version and scoring rules. Neurology 1993, 43: 2412–2414.

[4] Petersen RC, Smith GE, Waring SC, Ivnik RJ, Tangalos EG, Kokmen E. Mild cognitive impairment: Clinical characterization and outcome. Arch Neurol 1999, 56: 303–308.

[5] Feng F, Wang P, Zhao K, Zhou B, Yao H, Meng Q, *et al*. Radiomic features of hippocampal subregions in Alzheimer's disease and amnestic mild cognitive impairment. Front Aging Neurosci 2018, 10: 290.

[6] Alpert K, Kogan A, Parrish T, Marcus D, Wang L. The northwestern university neuroimaging data archive (NUNDA). Neuroimage 2016, 124: 1131–1136.

[7] Zhou B, Yao H, Wang P, Zhang Z, Zhan Y, Ma J, *et al*. Aberrant functional connectivity architecture in Alzheimer's disease and mild cognitive impairment: A whole-brain, data-driven analysis. Biomed Res Int 2015, 2015: 495375.

[8] Wang P, Zhou B, Yao H, Zhan Y, Zhang Z, Cui Y, *et al*. Aberrant intra- and inter-network connectivity architectures in Alzheimer's disease and mild cognitive impairment. Sci Rep 2015, 5: 14824.

[9] Zhang Z, Liu Y, Zhou B, Zheng J, Yao H, An N, *et al*. Altered functional connectivity of the marginal division in Alzheimer's disease. Curr Alzheimer Res 2014, 11: 145–155.

[10] Yao H, Zhou B, Zhang Z, Wang P, Guo YE, Shang Y, *et al*. Longitudinal alteration of amygdalar functional connectivity in mild cognitive impairment subjects revealed by resting-state FMRI. Brain Connect 2014, 4: 361–370.

[11] Guo Y, Zhang Z, Zhou B, Wang P, Yao H, Yuan M, *et al*. Grey-matter volume as a potential feature for the classification of Alzheimer's disease and mild cognitive impairment: An exploratory study. Neurosci Bull 2014, 30: 477–489.

[12] Zhou B, Liu Y, Zhang Z, An N, Yao H, Wang P, *et al*. Impaired functional connectivity of the thalamus in Alzheimer's disease and mild cognitive impairment: A resting-state fMRI study. Curr Alzheimer Res 2013, 10: 754–766.

[13] Yao H, Liu Y, Zhou B, Zhang Z, An N, Wang P, *et al*. Decreased functional connectivity of the amygdala in Alzheimer's disease revealed by resting-state fMRI. Eur J Radiol 2013, 82: 1531–1538.
[14] Wang P, Zhang X, Liu Y, Liu S, Zhou B, Zhang Z, *et al*. Perceptual and response interference in Alzheimer's disease and mild cognitive impairment. Clin Neurophysiol 2013, 124: 2389–2396.
[15] Zhang Z, Liu Y, Jiang T, Zhou B, An N, Dai H, *et al*. Altered spontaneous activity in Alzheimer's disease and mild cognitive impairment revealed by Regional Homogeneity. Neuroimage 2012, 59: 1429–1440.
[16] Wang D, Hu L, Xu X, Ma X, Li Y, Liu Y, *et al*. KIBRA and *APOE* gene variants affect brain functional network connectivity in healthy older people. J Gerontol A Biol Sci Med Sci 2019, 74: 1725–1733.
[17] Yan T, Wang W, Yang L, Chen K, Chen R, Han Y. Rich club disturbances of the human connectome from subjective cognitive decline to Alzheimer's disease. Theranostics 2018, 8: 3237–3255.
[18] Li S, Yuan X, Pu F, Li D, Fan Y, Wu L, *et al*. Abnormal changes of multidimensional surface features using multivariate pattern classification in amnestic mild cognitive impairment patients. J Neurosci 2014, 34: 10541–10553.
[19] Lu J, Li D, Li F, Zhou A, Wang F, Zuo X, *et al*. Montreal cognitive assessment in detecting cognitive impairment in Chinese elderly individuals: A population-based study. J Geriatr Psychiatry Neurol 2011, 24: 184–190.
[20] Guo Q, Sun Y, Yu P, Hong Z, Lv C. Norm of auditory verbal learning test in the normal aged in China community. Chin J Clin Psychol 2007, 15: 132–134.
[21] Hamilton M. A rating scale for depression. J Neurol Neurosurg Psychiatry 1960, 23: 56–62.
[22] Dozeman E, van Schaik DJF, van Marwijk HWJ, Stek ML, van der Horst HE, Beekman ATF. The center for epidemiological studies depression scale (CES-D) is an adequate screening instrument for depressive and anxiety disorders in a very old population living in residential homes. Int J Geriatr Psychiatry 2011, 26: 239–246.
[23] Petersen RC. Mild cognitive impairment as a diagnostic entity. J Intern Med 2004, 256: 183–194.
[24] McKhann G, Drachman D, Folstein M, Katzman R, Price D, Stadlan EM. Clinical diagnosis of Alzheimer's disease: Report of the NINCDS-ADRDA Work Group under the auspices of Department of Health and Human Services Task Force on Alzheimer's Disease. Neurology 1984, 34: 939–944.
[25] McKhann GM, Knopman DS, Chertkow H, Hyman BT, Jack CR Jr, Kawas CH, *et al*. The diagnosis of dementia due to Alzheimer's disease: Recommendations from the National Institute on Aging-Alzheimer's Association workgroups on diagnostic guidelines for Alzheimer's disease. Alzheimers Dement 2011, 7: 263–269.

[26] Petersen RC, Doody R, Kurz A, Mohs RC, Morris JC, Rabins PV, *et al*. Current concepts in mild cognitive impairment. Arch Neurol 2001, 58: 1985–1992.
[27] Choo IH, Lee DY, Youn JC, Jhoo JH, Kim KW, Lee DS, *et al*. Topographic patterns of brain functional impairment progression according to clinical severity staging in 116 Alzheimer disease patients: FDG-PET study. Alzheimer Dis Assoc Disord 2007, 21: 77–84.
[28] Wu Y, Zhang Y, Liu Y, Liu J, Duan Y, Wei X, *et al*. Distinct changes in functional connectivity in posteromedial cortex subregions during the progress of Alzheimer’s disease. Front Neuroanat 2016, 10: 41.
[29] Liu J, Zhang X, Yu C, Duan Y, Zhuo J, Cui Y, *et al*. Impaired parahippocampus connectivity in mild cognitive impairment and Alzheimer’s disease. J Alzheimers Dis 2016, 49: 1051–1064.
[30] Liu Y, Yu C, Zhang X, Liu J, Duan Y, Alexander-Bloch AF, *et al*. Impaired long distance functional connectivity and weighted network architecture in Alzheimer’s disease. Cereb Cortex 2014, 24: 1422–1435.
[31] He X, Qin W, Liu Y, Zhang X, Duan Y, Song J, *et al*. Abnormal salience network in normal aging and in amnestic mild cognitive impairment and Alzheimer’s disease. Hum Brain Mapp 2014, 35: 3446–3464.
[32] Song J, Qin W, Liu Y, Duan Y, Liu J, He X, *et al*. Aberrant functional organization within and between resting-state networks in AD. PLoS One 2013, 8: e63727.
[33] Yu E, Liao Z, Tan Y, Qiu Y, Zhu J, Han Z, *et al*. High-sensitivity neuroimaging biomarkers for the identification of amnestic mild cognitive impairment based on resting-state fMRI and a triple network model. Brain Imaging Behav 2019, 13: 1–14.
[34] Brueggen K, Grothe MJ, Dyrba M, Fellgiebel A, Fischer F, Filippi M, *et al*. The European DTI Study on Dementia - A multicenter DTI and MRI study on Alzheimer’s disease and Mild Cognitive Impairment. Neuroimage 2017, 144: 305–308.
[35] Gaser C, Dahnke R. CAT-a computational anatomy toolbox for the analysis of structural MRI data. Hbm 2016, 2016: 336–348.
[36] Gilmore AD, Buser NJ, Hanson JL. Variations in structural MRI quality significantly impact commonly used measures of brain anatomy. Brain Inform 2021, 8: 7.
[37] Fortin JP, Cullen N, Sheline YI, Taylor WD, Aselcioglu I, Cook PA, *et al*. Harmonization of cortical thickness measurements across scanners and sites. Neuroimage 2018, 167: 104–120.
[38] Burt JB, Helmer M, Shinn M, Anticevic A, Murray JD. Generative modeling of brain maps with spatial autocorrelation. Neuroimage 2020, 220: 117038.

[39] Viladomat J, Mazumder R, McInturff A, McCauley DJ, Hastie T. Assessing the significance of global and local correlations under spatial autocorrelation: A nonparametric approach. Biometrics 2014, 70: 409–418.
